# Functional Polarization of Liver Macrophages by Glyco Gold Nanoparticles

**DOI:** 10.1002/advs.202407458

**Published:** 2025-02-14

**Authors:** Jennifer Fernandez Alarcon, Patricia Perez Schmidt, Nicolo Panini, Francesca Caruso, Martina B. Violatto, Naths Grazia Sukubo, Alberto Martinez‐Serra, Charlotte Blanche Ekalle‐Soppo, Annalisa Morelli, Giulia Yuri Moscatiello, Chiara Grasselli, Alessandro Corbelli, Fabio Fiordaliso, Joe Kelk, Laura Petrosilli, Giuseppe d'Orazio, Ruth Mateu Ferrando, Ariadna Verdaguer Ferrer, Cristina Fornaguera, Luigi Lay, Stefano Fumagalli, Sandro Recchia, Marco P. Monopoli, Laura Polito, Paolo Bigini, Giovanni Sitia

**Affiliations:** ^1^ Department of Molecular Biochemistry and Pharmacology Istituto di Ricerche Farmacologiche Mario Negri IRCCS Via Mario Negri 2 Milano 20156 Italy; ^2^ Grup d'Enginyeria de Materials (GEMAT) Institut Químic de Sarrià (IQS) Universitat Ramon Llull (URL) Via Augusta 390 Barcelona 08017 Spain; ^3^ Istituto di Scienze e Tecnologie Chimiche “Giulio Natta” SCITEC‐CNR, Via G. Fantoli 16/15 Milano 20138 Italy; ^4^ Department of Oncology Istituto di Ricerche Farmacologiche Mario Negri IRCCS Via Mario Negri 2 Milano 20156 Italy; ^5^ Experimental Hepatology Unit Division of Immunology, Transplantation and Infectious Diseases IRCCS San Raffaele Scientific Institute Via Olgettina 58 Milano 20132 Italy; ^6^ School of Medicine and Surgery University of Milano‐Bicocca Piazza dell'Ateneo Nuovo 1 Milano 20126 Italy; ^7^ Department of Chemistry Royal College of Surgeons of Ireland RCSI St Stephens Green 123 Dublin Ireland; ^8^ Department of Neurosciences Istituto di Ricerche Farmacologiche Mario Negri IRCCS Via Mario Negri 2 Milano 20156 Italy; ^9^ Department of Organic Chemistry University degli Studi di Milano Via Golgi 19 Milano 20133 Italy; ^10^ Department of Analytical and Applied Chemistry Institut Químic de Sarrià (IQS) Universitat Ramon Llull (URL) Via Augusta 390 Barcelona 08017 Spain; ^11^ Department of Science and High Technology University of Insubria Via Valleggio 11 Como 22100 Italy

**Keywords:** glycans, gold nanoparticles, hepatic metastases, immunotherapy, primary biliary cholangitis

## Abstract

Macrophages are crucial drivers of innate immunity. Reprogramming macrophages to a restorative phenotype in cancer or autoimmune diseases can stop their cancer‐promoting activity or trigger anti‐inflammatory immunity. Glycans have emerged as key components for immunity as they are involved in many pathophysiological disorders. Previous studies have demonstrated that supraphysiological amounts of mannose (Man) or sialic acid (Sia) can inhibit tumor growth and stimulate differentiation of regulatory T cells. Man is known to affect glucose metabolism in glycolysis by competing for the same intracellular transporters and affecting macrophage polarization, whereas Sia alters macrophage differentiation via signaling through Siglec‐1. Herein, this work describes a macrophage targeting platform using gold nanoparticles (GNPs) functionalized with Man and Sia monosaccharides which exhibit high liver tropism. A single dose of glyco‐GNPs can convert macrophages to a restorative phenotype in two completely different immune environments. Man promotes tumor‐associated macrophages toward an antitumorigenic activity in a MC38 liver colorectal cancer model by secretion of TNF‐α, IL ‐1β, and IL ‐6 in the tumor microenvironment. However, in a proinflammatory environment, as observed in a mouse model of autoimmune disease, primary biliary cholangitis, Man impairs the production of TNF‐α, IL‐1β, Arg1, and IL‐6 cytokines. The results probe the dual role of Man in macrophage repolarization in response to the immune system. This study is a proof‐of‐concept that demonstrates that nanomedicine using specific glycans designed to target other immune cells such as myeloid cells, are a promising strategy not only against cancer but also against other pathologies such as autoimmune diseases.

## Graphical abstract

Macrophages play a critical role in several pathophysiological diseases by being involved in the activation of adaptive and innate immune responses. The repolarization of macrophages using gold nanoparticles functionalized with glycans for a more selective targeting toward a restorative phenotype leads to an amelioration of the events. These findings provide important insights for exploiting glycans to treat autoimmune disorders or cancer.

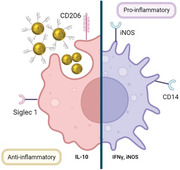

## 1 Introduction

Several glycans have been identified as mediating key molecules in pathophysiological events such as cancer or autoimmune diseases.^[^ ^1^, ^2^, ^3^ ^]^ Located on the outermost surface of cells, glycans are involved in diverse cellular processes such as cell adhesion, cell motility, and receptor activation.^[^ ^3^ ^]^ Glycans bind to specific glycan‐binding proteins which exist on the surface of endothelial cells lining the vasculature and immune cells.^[^ ^1^ ^]^ Alterations in glycosylation have been observed to promote tumor progression, due to dysregulation in cell proliferation and evasion of innate immunity.^[^ ^2^ ^]^

Macrophages are essential for innate immunity, acting as a first line of defense.^[^ ^4^ ^]^ Their plasticity and diversity allow them to adapt to environmental cues, actively participating in the immune response and resolution of pathological insults, which lead to tissue injury and promote regeneration.^[^ ^3^ ^]^ Originating from the bone marrow precursor, their monocyte‐macrophage differentiation depends on signals from damaged tissues, pathogens, or lymphocytes.^[^ ^5^, ^6^ ^]^ Macrophages are implicated in a variety of pathological conditions including inflammatory liver diseases, being recognized into two different states of polarized activation M1 or M2, offering two distinct cytokine profiles.^[^ ^7^ ^]^ M1 macrophages, or classically activated macrophages, can phagocyte pathogens and offer antitumor properties.^[^ ^7^ ^]^ In contrast, M2 macrophages have an anti‐inflammatory profile, promoting tumoral progression and tissue repair depending on the type of stimuli.^[^ ^8^ ^]^

The tumor microenvironment (TME) is a complex structure that evolves as the tumor progresses and promotes metastatic spread by engaging immune‐avoiding factors that include regulatory lymphocytes, as well as myeloid cells such as macrophages.^[^ ^4^ ^]^ New immunotherapeutic approaches have been approved for clinical use.^[^ ^9^, ^10^, ^11^, ^12^ ^]^ Antitumor treatments such as chimeric antigen receptor T‐cell therapy (CAR‐T‐cells) have become first‐ or second‐line treatments, offering more durable responses and even cures for many cancers, outcomes not achieved with standard chemotherapy.^[^ ^12^ ^]^ However, novel approaches to revive immune cells are underrepresented in current immunotherapies, complicating the application of these immunomodulatory agents in other physiopathological conditions such as autoimmune diseases.

Among the various glycan‐binding receptors present in the macrophage membrane, macrophage mannose receptor 1 (MR), also known as CD206, and sialic acid‐binding immunoglobulin I like lectin (Siglec‐1) recognized as CD169, are highly expressed and involved in macrophages stimuli.^[^ ^13^, ^14^, ^15^ ^]^ MR is a type I transmembrane protein that belongs to the C‐type lectin family, while Siglec‐1 is an I‐type lectin that contains 17 immunoglobulins (Ig) domains. Strategies to optimize drug uptake via these receptors may be beneficial.^[^ ^16^ ^]^

D‐Mannose (Man) has been shown to affect T cell function by promoting the generation of T regulatory cells (T_reg_) and suppressing T‐cell mediated immunopathologies in models of autoimmune diabetes type 1,^[^ ^17^, ^18^ ^]^ airway inflammation,^[^ ^17^, ^18^ ^]^ ulcerative colitis^[^ ^19^ ^]^ and lupus,^[^ ^20^ ^]^ mainly by interfering with the glucose metabolism, and reducing lactate formation from glucose.^[^ ^19^ ^]^ On the contrary, tumors have high avidity for glucose, and the use of Man has been shown to affect tumor cell growth by impairing glycolysis.^[^ ^21^ ^]^ Another glycan involved in immune homeostasis is sialic acid (Sia), which plays a role in the generation of T_reg_ via dendritic cells (DCs), stimulating an anti‐inflammatory response in macrophages and also, being overexpressed in cancer cells.^[^ ^22^, ^23^, ^24^ ^]^ The presence of Sia on the external side of the cell membrane contributes to the regulation of the innate immune system by interacting with Siglecs.^[^ ^16^, ^25^, ^26^ ^]^

Targeting these receptors with nanoparticles (NPs) combined with glycans can be of interest as a therapeutic approach for hepatic diseases such as autoimmune disorders characterized by pro‐inflammatory immune infiltrates^[^ ^20^, ^27^, ^28^ ^]^ or cancer‐recruiting protumoral myeloid cells with immunosuppressive activity.^[^ ^3^, ^8^ ^]^ The opportunity to functionalize nanomaterials with specific glycans, can enhance their efficacy exploiting the multivalent effect, and helping to favor the passage of active compounds to their targeting area, reducing off‐targeting in healthy organs. Here, we exploited glycosylated gold NPs (Glyco‐GNPs) functionalized with two different monosaccharides, Man and Sia, to re‐program macrophages into a restorative phenotype without disrupting immune homeostasis or causing a systemic toxicity. We found that the biodistribution of glyco‐GNPs in healthy mice after a single intravenous administration reached the liver and were internalized by liver resident sinusoidal macrophages, Kupffer cells (KCs), and liver sinusoidal endothelial cells (LSECs). Using two different murine animal models, recapitulating liver colorectal cancer (CRC) metastatic disease, or primary biliary cholangitis (PBC), characterized by opposite intrahepatic macrophage polarization, to characterize the effect of glycans in repolarizing macrophages with two completely different profiles. Our results demonstrate that both glyco‐GNPs can alter the composition of tumor‐associated macrophages (TAMs) in the TME into a pro‐inflammatory population, rendering the tumor immunogenic. However, Man‐GNPs induced a higher antitumor activity in metastatic liver. We also found that Man‐GNPs have a dual role, being able to stimulate pro‐inflammatory macrophages in PBC into anti‐inflammatory. Based on this finding, these results demonstrated that the exploitation of glycans for immunotherapies as Man, may modulate myeloid cells in TME or in an autoimmune environment representing a promising alternative to standard therapies.

## 2 Results

### 2.1 Synthesis and Physicochemical Characterization of (glyco‐)GNPs

**Figure 1:**
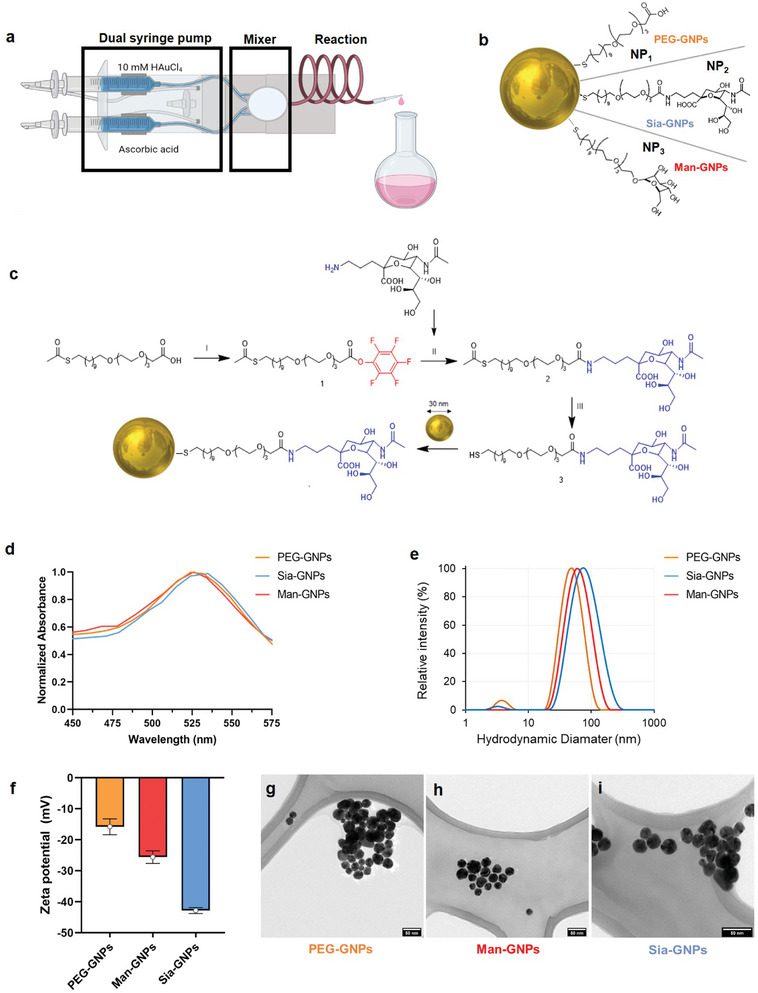
Generation and characterization of glyco‐GNPs to target hepatic macrophages. a) In a typical protocol, one syringe is loaded with HAuCl_4_ solution (0.015 × 10^−3^ m) and the other with AA solution (0.004 × 10^−3^ m). When both solutions are mixed, the reduction of HAuCl_4_ to metallic gold (Au^0^) occurs in the coil. Image created with Biorender. b) Schematic diagram of the chemical composition of GNPs using either EG_6_C_11_SH (PEG), Man or Sia. c) Synthetic scheme of Sia‐GNPs. I) PFP, DMC, EDC, RT, 18 h; II) 3‐Aminopropyl‐*N*‐acetylneuraminic acid dissolved in DMF, DIPEA, DMC, RT, 36 h; III) MeOH/MeONa, pH = 9, RT, 1 h. d) UV–vis spectrum showing a small bathochromic shift of the SPR band (absorption peak around 529 nm for PEG ‐GNPs, 526 nm for Man‐GNPs and 527 nm for Sia‐GNPs). e) Dynamic light scattering (*d*_h_) and f) zeta potential (ζ) of 47 ± 1 nm and *ζ* = −15.8 ± 5.7 mV for PEG‐GNPs; 65 ± 2 nm and *ζ* = −26 ± 4.5 mV for Man‐GNPs; 70 ± 3 nm and *ζ* = −43 ± 2.2 mV for Sia‐GNPs. g–i) Representative TEM image of g) PEG‐GNPs (gold core size of 29.9 ± 2.6 nm), h) Man‐GNPs (30.6 ± 2.7 nm) and i) Sia‐GNPs (31.0 ± 3.5 nm). Scale bar = 50 nm.

To study the effect of the different glycans on the different hepatic cell populations, we synthesized spherical GNPs with a diameter of about 30 nm and functionalized them with three different types of ligands. All GNPs were functionalized with an amphiphilic linker, 11‐(methylcarbonylthio)‐undecyl‐tri(ethylene glycol) acetic acid (EG_6_C_11_SH), which has a similar but shorter structure compared to the biocompatible polymer poly(ethylene) glycol (PEG) and then, with two different glycans, Man or Sia. All NPs were synthesized using a one‐step protocol, based on a microfluidic system^[^ ^22^, ^29^ ^]^ reducing tetrachloroauric acid (HAuCl_4_) to metallic gold (Au^0^) by using ascorbic acid (AA) as a reducing agent at room temperature (RT) and in an aqueous environment. This method allows rapid and efficient preparation of highly reproducible NPs without the need for toxic templating agents such as cetyltrimethylammonium (CTAB) (**Figure** **1**a).

To functionalize the GNPs with the monosaccharides, we had to develop a synthetic methodology to conjugate the aminoglycans directly onto the polymer ligands in an aqueous solution. The synthetic strategy developed for Sia‐GNPs is shown in Figure 1b,c and the strategy for Man‐GNPs is described in Figure S1 (Supporting Information). The RAFT agent, pentafluorophenol (PFP), was used to target EG_6_C_11_SH. The polymer was characterized by ^1^H and ^19^F NMR (Figure S2, Supporting Information).

Next, the polymer (PFP‐EG_6_C_11_SH) was reacted with the negatively charged 3‐aminopropyl‐N‐Acetylneuraminic acid displacing the PFP group. Since the *α*‐ and *β*‐ ^1^H NMR and ^13^C NMR signals from the Sia monomer overlap with those from the polymer, the presence of an amide bond was confirmed by a peak ≈1.9 ppm (Figure S3, Supporting Information) and by the appearance of *α‐* and *β‐*monomers signals by TLC. The glycosylated polymers were purified using a size exclusion chromatography, the thiol group was deprotected and then, used in the next step to coat 30 nm GNPs. The glycopolymers were mixed with the GNPs dispersed in pure water (Figure 1b). Following this, excess glycan and polymer were removed by centrifugation and resuspension cycles.

UV–vis spectroscopy and DLS were used to characterize the glyco‐GNPs (Figure 1d–f). There was a small bathochromic shift of the SPR band (Figure 1d), the hydrodynamic diameter of the NPs rose to 47 nm for PEG‐GNPs, to 65 nm for Man‐GNPs, and 70 nm for Sia‐GNPs, supportive of glycopolymer grafting to the surface (Figure 1e), and an increase in the zeta potential (*ζ*) toward a more negative overall net charge (Figure 1f). A representative product of GNPs synthesis is shown in Figure 1g–i (Figure S4, Supporting Information).

### 2.2 Glyco‐GNPs Hepatic Biodistribution

Innate immune system's response toward a stimulation is rapid, occurring within the first hours after NP administration.^[^ ^30^, ^31^ ^]^ To investigate changes in hepatic immune‐responsive cells we selected an early time point of 4 h after treatment. We injected 2 × 10^11^ NPs per healthy mouse to evaluate the effect of glyco‐GNPs on different hepatic cell populations.

**Figure 2:**
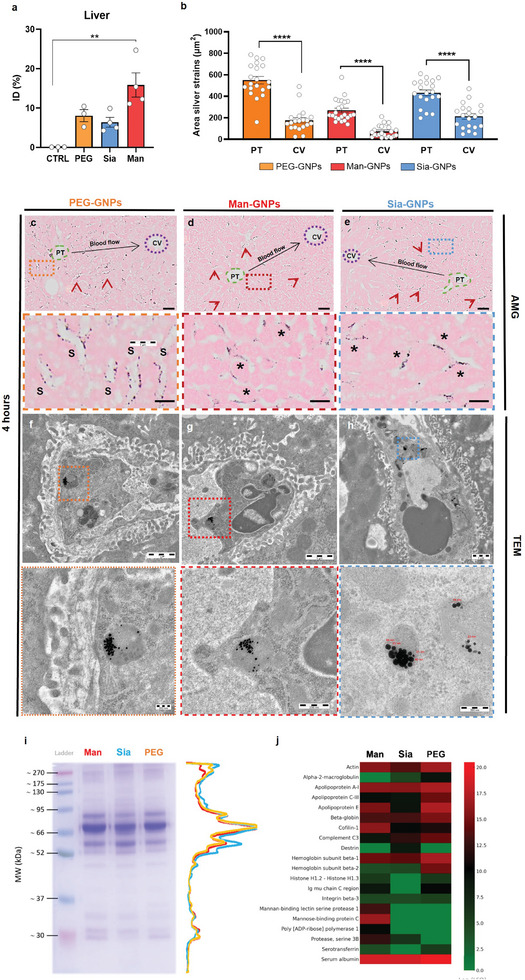
Biodistribution of glyco‐GNPs in liver and biomolecular interaction. a) Quantification of glyco‐GNPs by ICP‐MS in healthy mice treated after 4 h. Data are presented as mean ± SEM of *n* = 4 mice, except PEG *n* = 3. *p* values were determined by one‐way ANOVA with Bonferroni's correction ^*^ *p* < 0.05 and ^**^ *p* < 0.01. b) Scatter plot comparing the areas surrounding PT versus CV of PEG‐GNPs, Man‐GNPs, and Sia‐GNPs. Data are presented as mean ± SEM of *n* = 10 images per condition. *p* values were determined by two‐way Student's *t*‐test ^****^ *p* < 0.0001. c–e) Representative micrographs of liver after AMG staining from mice treated with 30 nm glyco‐GNPs with c) PEG‐GNPs and d) Man‐GNPs or e) Sia‐GNPs euthanized after 4 h. Red arrows show black‐stained glyco‐GNPs inside hepatic cells. Black arrows show GNP circulation in the liver. Green dotted lines show GNPs circulating from portal triad (PT) and purple dotted lines show the central vein (CV). Scale bars for AMG images = 100 µm. Magnifications of AMG images are represented on the lower side showing GNPs interacting with sinusoids (S) and internalized inside hepatic cells (*). Scale bars for AMG magnifications = 20 µm. f–h) Representative TEM images of ultrastructural localization of glyco‐GNPs inside KCs of healthy mice treated with f) PEG‐GNPs, g) Man‐GNPs, or h) Sia‐GNPs after 4 h. Scale bars for TEM images (onset) = 1 µm; Scale bar (inset) = 200 nm. i) Blue Coomassie‐stained SDS‐PAGE and its corresponding densitometry analysis for the three different GNPs. j) Protein MS heatmap of the most abundant proteins in the HC of the three GNPs, represented as Log2(LFQ). The measurements were performed in triplicates.

The administrated dose was chosen because it is comparable to other studies and did not result in toxicity.^[^ ^32^ ^]^ Biodistribution in the liver was assessed using inductively coupled plasma mass spectrometry (ICP‐MS) (**Figures** **2**a and S5, Supporting Information), silver staining (AMG) (Figures 2b–e, S6 and S7, Supporting Information) and, transmission electron microscopy (TEM) (Figures 2f–h and S8, Supporting Information). ICP‐MS revealed high liver tropism for Man‐GNPs (20% of injected dose; ID), followed by PEG‐GNPs (10% ID) and Sia‐GNPs (7% ID). No GNPs were detected in kidneys and lungs (Figure S5, Supporting Information). Only glycan‐coated GNPs were found in spleen, likely due to the high amount of macrophages present in the red pulp (Figure S9, Supporting Information).

Quantification of AMG signal near the portal triad (PT) and central vein (CV) indicated faster internalization of Sia‐GNPs and PEG‐GNPs in sinusoids bordering the PT compared to Man‐GNPs (Figure 2b). The appearance of black elongated bodies throughout the liver parenchyma confirmed the entry and internalization of the glyco‐GNPs in hepatic cells such as KCs and LSECs during the first hours post‐injection (Figure 2c–e). TEM confirmed glyco‐GNPs within lysosomes of KCs and LSECs with no signs of NPs in hepatocytes or other cells in the liver parenchyma (Figure 2f–h, Figure S8, Supporting Information). The internalization did not affect the ultrastructural organization of KCs or LSECs showing perfectly preserved organelles such as endoplasmic reticulum, Golgi apparatus, and nucleus.

To understand the behavior of the different GNPs in the nanoscale, we evaluated their bio‐nano interactions through physicochemical and biomolecular characterization (Figure 2i,j, Figure S10, Supporting Information). Differential centrifugal sedimentation (DCS) showed that pristine Man‐GNPs displayed a narrow size distribution similar to PEG‐GNPs, indicating a monodisperse particle population, however, Sia‐GNPs, show a broader and more complex size distribution, suggesting a polydisperse population. Upon exposure to plasma and hard corona (HC) formation, all NP types show a size decrease. Man‐GNP and PEG‐GNPs maintained good colloidal stability, while Sia‐GNPs exhibited a secondary peak, suggesting lower colloidal stability and possible agglomeration. SDS‐PAGE analysis revealed similar protein patterns among the NPs, with variations in peak intensities, particularly four protein bands in Mw range of 90–52 kDa and duplet bands of 30 kDa were equally detected in all samples. However one band had a lower intensity in the Sia sample.

Mass spectrometry was carried out to identify the top abundant corona protein and also to evaluate semiquantitative changes by comparing the LFQ intensity across the GNP samples (Figure 2j). The heatmap shows how serum albumin (66 kDa), ApoA1 (30 kDa), and ApoE (35k Da) are the predominant proteins identified by mass spectrometry, consistent with the SDS‐PAGE protein pattern.^[^ ^33^ ^]^ The lower presence of immunoglobulins and serotransferrin in the heatmap for Sia aligns with the results from the SDS‐PAGE, corresponding to the band below 95 kDa marker which is lighter compared to Man and PEG.

Interestingly, mannose‐binding lectin (MBL), which has an affinity toward Man, fucose, and *N*‐acetylglucosamine, was enriched in the Man‐GNPs, indicating that Man remains biologically active and capable of binding to MBL even after corona formation.^[^ ^34^, ^35^, ^36^ ^]^ Consequently, these particles exhibit active targeting and are particularly effective in binding specific proteins such as MBL and Mannan‐binding lectin serine protease 1 (MASP‐1). These proteins are associated with the lectin binding pathway which is the innate immunological response that is generally triggered after the blood plasma is exposed to pathogens. Elevated levels of MBL in blood are considered a useful biomarker for stroke, as they are associated with inflammation and vascular damage, which are key factors in stroke development and progression.^[^ ^37^ ^]^

### 2.3 Intrahepatic Cellular Interaction

**Figure 3:**
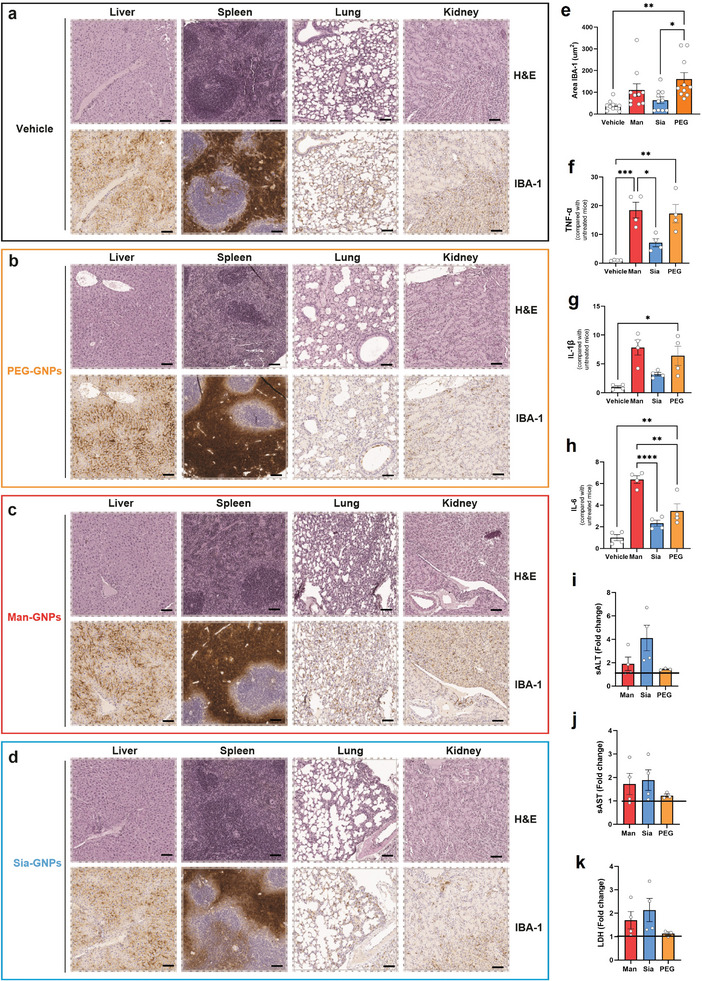
Effect of a single dose administration of glyco‐GNPs on the liver of healthy mice. a–d) H&E and IBA‐1 staining in livers, spleens, lungs, and kidneys from mice treated with glyco‐GNPs and sacrificed after 4 h. Scale bars = 50 µm. e) Quantification of IBA‐1^+^ cells in hepatic tissue. f–h) rt‐PCR of cytokines expressed in hepatic tissue of healthy mice treated glyco‐GNPs. Steady‐state levels of mRNAs for all target genes were normalized to nontreated healthy mice (Vehicle). f) Gene expression of TNF‐α mRNAs, g) IL‐1β mRNAs, and h) IL‐6 mRNAs. Data are presented as mean ± SEM of *n* = 4 mice. *p* values were determined by one‐way ANOVA with Bonferroni's correction ^*^ *p* < 0.05, ^**^ *p* < 0.01, ^***^ *p* < 0.001, and ^****^ *p* < 0.0001. i–k) Hepatic transaminase levels in serum. Fold change of i) sALT, j) sAST, and k) sLDH measured after 4 h in groups of mice injected with Man‐GNPs, Sia‐GNPs, and PEG‐GNPs compared to untreated controls (*n* = 4, for each group except PEG *n* = 3). Data are presented as mean ± SEM. No significant difference was determined *p* > 0.05.

Our initial study provided an organ‐level view of the GNP biodistribution, then, we proceeded to analyze at a sub‐organ level. Histological evaluation of liver, spleen, lungs, and kidneys by H&E staining showed no changes in the cell or tissue morphology (**Figure** **3**a–d). However, an increase in the leukocytes infiltrating areas next to the PT was observed by H&E. IBA‐1 staining revealed that these microgranulomas colocalized with macrophages, suggesting an acute inflammatory response to the glyco‐GNPs. Quantification of IBA‐1 in the hepatic tissues demonstrated a significant increase in IBA‐1^+^ cells for all GNPs compared to the vehicle group. However, only significant difference was found in PEG‐GNPs, due to an unspecific immune system activation (Figure 3e). Gene expression of the pro‐inflammatory cytokines TNF‐α, IL‐1β, and IL‐6 in the liver indicated upregulation at the mRNA levels 4 h after glyco‐GNPs injection (Figure 3f–h).

Man‐GNPs and PEG‐GNPs induced a higher pro‐inflammatory response in the hepatic tissue compared to Sia‐GNPs (Figure 3f–h). No significant differences were observed in other markers, such as iNOS, Arg1, IL‐10, and TGF‐β (Figure S11, Supporting Information). Histological evidence was confirmed by hematochemistry: the enzymatic levels for the serum transaminase released by liver cells such as alanine transaminase (sALT), aspartate transaminase (sAST), or lactate dehydrogenase (sLDH) did not reveal any significant toxicity (Figure 3i–k).

**Figure 4:**
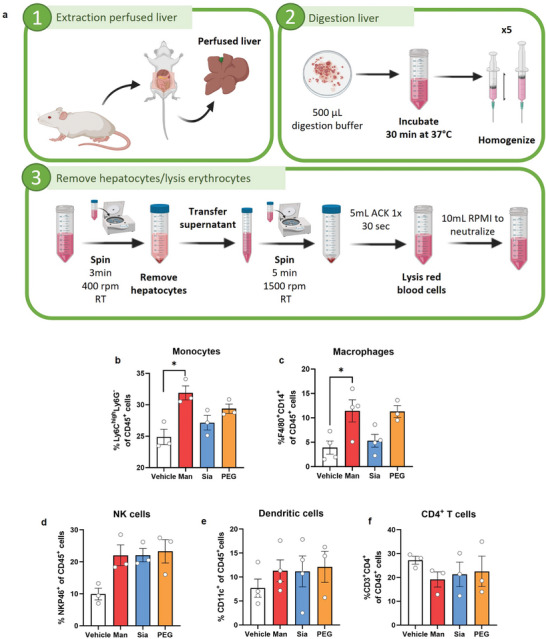
Effect of a single dose administration of glyco‐GNPs on the hepatic cell populations of healthy mice. a) Experimental scheme of the digestion of perfused liver in mice: 1) Extraction of the perfused liver through vena cava cannulation, 2) enzymatic digestion of the liver and homogenization, and 3) density removal of hepatocytes and erythrocyte lysis. Image created with Biorender. b) Quantification of hepatic cell populations of Ly6C^high^Ly6G^−^ monocyte‐macrophage cells. Data are presented as mean ± SEM of *n* = 3 mice. *p* values were determined by one‐way ANOVA with Bonferroni's correction ^*^ *p* < 0.05. c–f) Quantification of viable hepatic leukocytes c) CD45^+^ F4/80^+^ CD14^+^ macrophages, d) CD45^+^ NKP46^+^ NK cells, e) CD45^+^ CD11c^+^ dendritic cells (DCs), and f) CD45^+^ CD3^+^ CD4^+^ T cells. Data are presented as mean ± SEM of *n* = 3 mice. *p* values were determined by one‐way ANOVA with Bonferroni's correction ^*^ *p* < 0.05.

To demonstrate the specific targeting properties of glycans, we isolated liver cells from treated mice with glyco‐GNPs (**Figure** **4**a) and assessed the effects on various liver cell types with flow cytometry (gating strategy in Figure S12, Supporting Information). As expected, Man‐GNPs significantly increased the Ly6C^high^ Ly6G‐ monocyte‐macrophage populations in the liver, whereas PEG‐GNPs and Sia‐GNPs showed no significant difference compared to the vehicle group (Figure 4b). Indeed, the presence of Man‐GNPs also significantly increased the expression of F4/80^+^CD14^+^ in activated macrophages (Figure 4c). Although high levels were observed for PEG‐GNPs, Sia‐GNPs had no effect showing similar levels to untreated mice.

Four hours post‐GNP injection, we found an increase in hepatic cell populations including NKP46^+^ NK cells, CD3^+^NKP46^+^ NKT cells, Ly6C^high^ Ly6G^high^ neutrophils and CD11c^+^ DCs, while CD4^+^ T cells decreased (Figure 4d–f, Figure S13, Supporting Information). The activation of cytotoxic NK cells depends on the cytokine microenvironment, and interactions with other immune cells such as macrophages, DCs and T cells.^[^ ^38^, ^39^, ^40^ ^]^ An increase in proinflammatory Ly6C^high^ Ly6G^−^ cells in the liver triggered a cascade effect, raising NKP46^+^ NK cell levels (Figure 4d) and conventional CD11c^+^ DCs specialized in antigen presentation to T cells (Figure 4e), facilitating the activation of CD4^+^ and CD8^+^ T cells.^[^ ^18^, ^20^ ^]^ However no lymphocyte activation (T or B cells) was observed (Figure 4f, Figure S13, Supporting Information). No significant difference was observed in other hepatic cell types such as B220^+^ B cells (Figure S13, Supporting Information). An increased expression of F4/80^+^CD206^+^ cells and F4/80^+^CD169^+^ cells in livers treated with glyco‐GNPs demonstrated an immune activation (Figure S14, Supporting Information).

Overall, we found that glyco‐GNPs increased the number of hepatic macrophages, which were significantly affected by the glycan's presence on the GNP surface. Therefore, we decided to test the capacity of glycans to functionally polarize liver macrophages in mouse models of liver disease characterized by two completely differentially polarized macrophage populations.

### 2.4 Glyco‐GNPs can Activate Macrophages in Metastatic Liver

**Figure 5:**
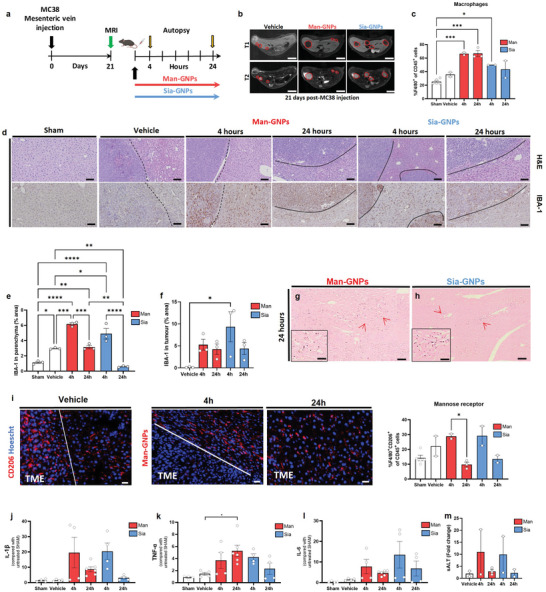
Activation of hepatic macrophages by a single administration of glyco‐GNPs in tumor‐bearing mice (CRC). a) A total of 8‐ to 10‐week‐old male C57B/6J mice were injected in the superior mesenteric vein with 7 × 10^4^ MC38 CRC cells and 21 days later the intrahepatic CRC bourden was analyzed by MRI analysis. Mice were intravenously injected with either Man‐GNPs, Sia‐GNPs or vehicle. An additional group of tumor‐free sham mice was used as inner control for FACS analysis. b) Representative MRI images of mice liver intramesenterically injected with MC38 CRC cells. Dashed red lines highlight CRC liver metastases characterized as hypointense and slightly hyperintense regions in T1‐ and T2‐weighted sequences, respectively. Scale bars = 5 mm. c) Liver NPCs were isolated and leukocyte population was quantified by FACS, Ly6C^high^Ly6G^−^ monocyte‐macrophage cells. Data are presented as mean ± SEM of *n* = 3 mice. *p* values were determined by one‐way ANOVA with Bonferroni's correction ^*^ *p* < 0.05 and ^**^ *p* < 0.01. d) H&E and IBA‐1 staining in livers treated with glyco‐GNPs after 4 and 24 h. Dashed lines indicate the tumor borders in the liver parenchyma. Scale bars = 50 µm. e,f) Quantification of IBA‐1^+^ cells in e) hepatic tissue and f) tumors. Data are presented as mean ± SEM of *n* = 3 mice. *p* values were determined by one‐way ANOVA with Bonferroni's correction ^*^ *p* < 0.05, ^**^ *p* < 0.01, ^***^ *p* < 0.001, and ^****^ *p* < 0.0001. g,h) Representative liver micrographs stained with AMG from mice treated with g) Man‐GNPs or h) Sia‐GNPs euthanized 24 h after the treatment. Red arrows show silver‐stained glyco‐GNPs inside hepatic cells. Scale bars = 100 µm (large image) and 20 µm (small insets). i) CD206 immunostaining in livers treated with glyco‐GNPs after 4 h. In blue nuclei and in red CD206^+^ cells. Scale bars = 25 µm. Quantification of hepatic cell populations of CD45^+^F4/80^+^CD206^+^ monocyte‐macrophage cells. Data are presented as mean ± SEM of *n* = 2 mice. *p* values were determined by one‐way ANOVA with Bonferroni's correction ^*^ *p* < 0.05. j) Gene expression of IL‐1β mRNAs, k) TNF‐α mRNAs, and l) IL‐6 mRNAs. Data are presented as mean ± SEM of *n* = 2 mice. No significant difference was determined *p* > 0.05. m) Hepatic transaminase levels in serum. Fold change of sALT measured after 4 and 24 h in mice injected with glyco‐GNPs. Data are presented as mean ± SEM of *n* = 2 mice. No significant difference was determined *p* > 0.05.

Liver metastases of CRC are associated with the worst prognosis of patients and high mortality despite the improvement of current therapeutic approaches. Therefore, new and more effective strategies to improve therapies for CRC are urgently needed. Liver metastatic growth relies on complex and diverse events, which include the recruitment of myeloid cells and macrophages with immunosuppressive and tumor promoting functions.^[^ ^41^ ^]^ In addition, tumors have a high requirement for glucose, and glycans such as Man have been shown to specifically block glucose transport within cells, thereby reducing tumor growth and promoting cell death.^[^ ^21^ ^]^ To investigate whether glyco‐GNPs decorated with Man and other glycans can functionally polarize liver macrophages, we administered a single intravenous injection of glyco‐GNPs to mice. In order to obtain a mouse model of CRC, immune competent C57B/6J mice were mesenterically challenged with the syngeneic MC38 CRC cell line, which has been recently reported to bear typical mutational and immunological features, of hypermutated MSI human tumors.^[^ ^42^ ^]^ The MC38 CRC cell line was injected at a known dose of 7 × 10^4^ cells per mouse (**Figure** **5**a). Contrast‐enhanced magnetic resonance imaging (MRI) probed the formation of the metastatic tumors in the liver 21 days after challenge (Figure 5b, dashed red lines). Mice were then treated with Man‐GNPs or Sia‐GNPs and euthanized 4 and 24 h later to study the effect on hepatic macrophages. This experimental setting may mimic the vascular spreading of CRC cells and, represents a valuable model of post‐resection metastatic recurrence in the liver in immunocompetent hosts.^[^ ^43^ ^]^

Flow cytometry analysis of hepatic non‐parenchymal cells (NPCs) from the glyco‐GNPs‐treated mice showed that both monosaccharides increased the amount of Ly6C^high^ Ly6G^−^ monocyte‐macrophage cell populations in the metastatic liver, but only Man‐treated mice showed a significant increase (Figure 5c). Histological evaluation of the metastatic hepatic tissues by H&E confirmed increased cell infiltration in the liver parenchyma and surrounding TME (Figure 5d). Interestingly, IBA‐1 staining demonstrated an increased amount of macrophages in the liver parenchyma and TME of glyco‐GNPs treated mice. Man‐GNP‐treated mice highlighted the presence of two distinct macrophage morphologies in the hepatic parenchyma: elongated at 4 h, as it was observed in sham mice similarly to normal KCs, and rounded at 24 h, indicative of a pro‐inflammatory phenotype^[^ ^44^, ^45^ ^]^ (Figure 5e,f).

To follow the biodistribution of glyco‐GNPs in the metastatic liver, we used AMG, which showed the appearance of black spots in round bodies within the TME, confirming the GNP‐specific targeting of hepatic macrophages especially in the TME (Figure 5g,h). Immunostaining for YM1^+^ cells, a marker for anti‐inflammatory cells, showed TAMs only in the TME of mice treated with Man‐GNPs (Figure S15, Supporting Information). In contrast, YM1^+^ cells were detected all over the liver parenchyma and near vessels in Sia‐GNPs and vehicle‐treated mice, indicating that NPs were unable to get into the TME, leaving TAMs in these areas.

The expression of other anti‐inflammatory receptors such as F4/80^+^CD206^+^ (Figure 5i) and F4/80^+^CD169^+^ (Figures S16 and S17, Supporting Information) confirmed a significant decrease over time in CD206^+^ cells and CD169^+^ cells in mice treated with Man‐GNPs. In these mice CD206^+^ cells were found near the TME 4 h post‐injection and inside the TME after 24 h (Figure 5i). In vehicle mice and Sia‐GNPs‐treated mice, CD206^+^ cells were distributed throughout the whole liver parenchyma (Figure 5i, Figure S17, Supporting Information).

TAMs produce anti‐inflammatory cytokines such as IL‐10 and TGF‐β, which inhibit tumoricidal lymphocytes, increasing regulatory lymphocyte populations. However, no significant difference was observed in IL‐10 or TGF‐β mRNA levels (Figure S18, Supporting Information). Gene expression of pro‐inflammatory cytokines expressed in the liver such as TNF‐α, IL‐1β, and IL‐6 was increased at the mRNA levels after 4 and 24 h from glyco‐GNPs injection(Figure 5j–l). Both glyco‐GNPs increased pro‐inflammatory cytokines expression in the metastatic hepatic tissue compared to the vehicle. Other markers such as iNOS also increased with both glyco‐GNPs (Figure S18, Supporting Information), while Arg1 was downregulated only in mice treated with Man‐GNPs (Figure S18, Supporting Information). An increase in the transaminase levels (10‐fold for sALT and LDH, and a 7‐fold for sAST) 4 h post‐GNP injection confirmed immunostimulation in the metastatic liver. The elevated hepatic enzymes levels may reflect cancer cell lysis due to macrophages polarization to a pro‐inflammatory state (Figure 5m, Figure S16, Supporting Information).

TME is a complex structure that evolves with tumor progression to promote metastatic spread. It involves myeloid cells, divided in different populations of macrophages known as TAMs and myeloid‐derived suppressor cells (MDSCs),^[^ ^46^ ^]^ and lymphocytes with regulatory functions.

**Figure 6:**
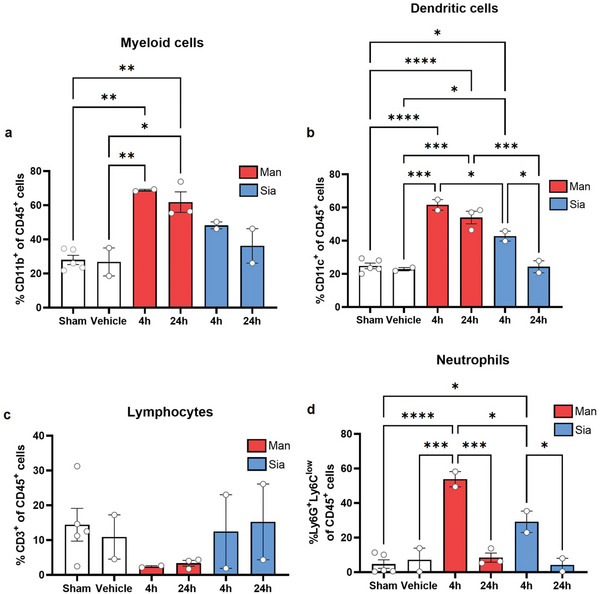
Effect of a single dose administration of glyco‐GNPs on hepatic cell populations in tumor bearing mice of CRC with hepatic metastatis. a–d) Quantification of viable hepatic cell populations a) CD45^+^CD11b^+^ myeloid cells, b) CD45^+^CD11c^+^ DCs, c) CD45^+^CD3^+^ T cells and d) Ly6C^high^Ly6G^low^ neutrophils. Data are presented as mean ± SEM of *n* = 2 mice. *p* values were determined by one‐way ANOVA with Bonferroni's correction ^*^ *p* < 0.05, ^**^ *p* < 0.01, ^***^ *p* < 0.001, and ^****^ *p* < 0.0001.

TAMs differentiate from myeloid cells depending on the kinetics of tumor growth. In treated metastatic livers a significant increase in MDSCs (CD11b^+^) was observed along with CD11c^+^ DC cells (**Figure** **6**a,b). However, Man‐GNPs had an inhibitory effect on CD3^+^ lymphocytic cells (Figure 6c). Other cell types such as Ly6G^+^Ly6^low^ neutrophil cells had a significant increase at earlier time points (Figure 6d). No significant difference was observed in other cell populations such as NKP46^+^ NK cells or CD4^+^ T cells (Figure S16, Supporting Information).

### 2.5 Man‐GNPs Repolarization of Macrophages in ARE Del^−/−^ Mice

Aberrant activation of pro‐inflammatory myeloid cells and sustained production of inflammatory cytokines, such as IFN‐γ, play a crucial role in the pathology of PBC.^[^ ^47^, ^48^ ^]^ To explore the role of Man in immune cell activation and its potential to repolarize macrophages toward a restorative phenotype in autoimmune diseases, we evaluated the effects of a single administration of Man‐GNPs in ARE Del^−/−^ mice model.

**Figure 7:**
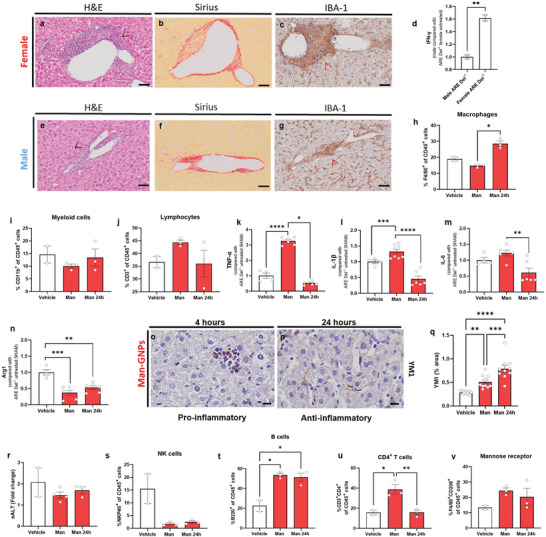
Re‐polarization of hepatic macrophages by a single administration of Man‐GNPs in murine model of primary biliary cholangitis (PBC). a–c) Histological evaluation of PBC model, ARE Del^−/−^ mice nontreated. Tissue sections of liver from ARE Del^−/−^ mice 18 weeks old female, with three different histological stains: a) H&E, nuclei (blue) and extracellular matrix and cytoplasm (pink). Black arrows show inflammatory foci in hepatic tissue. b) Sirius red, tissue (yellow) and collagen fibers (red) surrounding hepatic veins and sinusoids and c) IBA‐1, nuclei (blue), and macrophages (brown). Red arrows show macrophage infiltrations inside inflammatory foci in hepatic tissue. Scale bars = 50 µm. d) Gene expression of hepatic IFN‐γ mRNAs in ARE Del^−/−^ mice nontreated. Data are presented as mean ± SEM of *n* = 2 mice. *p* values were determined by one‐way ANOVA with Bonferroni's correction ^**^ *p* < 0.01. e–g) Representative micrographs of liver from a male ARE Del^−/−^ mice 18 weeks old nontreated stained with: e) H&E, black arrows show inflammatory foci in hepatic tissue. f) Sirius red, and g) IBA‐1, red arrows show macrophage infiltrations inside inflammatory foci in hepatic tissue. Scale bars = 50 µm. h–j) Quantification of hepatic cell populations of h) Ly6C^high^Ly6G^−^ monocyte‐macrophage cells, i) CD45^+^ CD11b^+^ myeloid cells, and j) CD45^+^ CD3^+^ lymphocytes. Data are presented as mean ± SEM of *n* = 3 mice. *p* values were determined by one‐way ANOVA with Bonferroni's correction ^*^ *p* < 0.05. k–n) Gene expression of livers treated with Man‐GNPs k) TNF‐α mRNAs, l) IL‐1β mRNAs, m) IL‐6 mRNAs, and n) Arg1 mRNAs. Data are presented as mean ± SEM of *n* = 3 mice. *p* values were determined by one‐way ANOVA with Bonferroni's correction ^*^ *p* < 0.05, ^**^ *p* < 0.01, ^***^ *p* < 0.001, and ^****^ *p* < 0.0001. o,p) Representative micrographs of liver from mice treated with Man‐GNPs euthanized after o) 4 h (pro‐inflammatory morphology) and p) 24 h (anti‐inflammatory morphology) by YM1 staining. Nuclei (blue) and macrophages (brown). Scale bars = 20 µm. q) Quantification of YM1^+^ cells in hepatic tissue. Data are presented as mean ± SEM of *n* = 10 images/condition. *p* values were determined by one‐way ANOVA with Bonferroni's correction ^**^ *p* < 0.01, ^***^ *p* < 0.001, and ^****^ *p* < 0.0001. r) Hepatic transaminase levels in serum. Fold change of sALT measured after 4 and 24 h in groups of mice injected with Man‐GNPs. Data are presented as mean ± SEM of *n* = 3 mice. No significant difference was determined *p* > 0.05. s–v) Quantification of viable hepatic cell populations s) CD45^+^NKP46^+^ NK cells, t) CD45^+^B220^+^ B cells, u) CD45^+^CD3^+^CD4^+^ T cells, and v) CD45^+^F4/80^+^CD206^+^ monocyte‐macrophage cells. Data are presented as mean ± SEM of *n* = 3 mice. *p* values were determined by one‐way ANOVA with Bonferroni's correction ^*^ *p* < 0.05 and ^**^ *p* < 0.01.

The overexpression of IFN‐γ in ARE Del^−/−^ mice, due the deletion of a portion of the adenylate uridine‐rich element (ARE) of IFN‐γ 3′‐untranslated region, causes the constant production of IFN‐γ and a mild autoimmune manifestation similar to PBC.^[^ ^20^ ^]^ This model has similarities to human PBC such as liver histology, antimitochondrial antibody (AMA) production and elevated serum total bile acid levels, with features predominantly found in female mice.^[^ ^47^ ^]^ Histological evaluation by H&E in vehicle ARE Del^−/−^ confirmed female predominance (**Figure** **7**). Both male and female mice at 18 weeks showed PT inflammation, but females exhibited larger and more numerous inflammatory foci, higher epithelioid granulomas, focal necrosis, and bile duct damage with infiltrations surrounding bile ducts, lobules, and the PT (Figure 7a–e). Increased collagen fibers were also observed in the surrounding areas of the vessels with some bridging from portal to portal veins (Figure 7b–f). IBA‐1 staining revealed extensive macrophage infiltration from hepatic lobules to PT (Figure 7c–g).

Consistent with histology, intrahepatic IFN‐γ gene expression demonstrated female predominance in ARE Del^−/−^ mice (Figure 7d). To follow the biodistribution of Man‐GNPs in PBC mice, AMG in the hepatic tissues of treated mice probed the appearance of elongated black bodies typical of KCs (Figure S19, Supporting Information). Man‐GNPs significantly increased the amount of monocyte‐macrophage cell populations (Ly6C^high^ Ly6G^−^) in the liver (Figure 7h), meanwhile no significant difference was observed for CD11b^+^ myeloid cells or CD3^+^ lymphocytes (Figure 7i,j). During chronic diseases such as PBC, it is normal to observe T cell exhaustion causing a poor effector function (Figure 7j). Gene expression of TNF‐α, IL‐1β, IL‐6, and Arg1 were significantly decreased at the mRNA levels after 24 h of Man‐NPs treatment in ARE Del^−/−^ mice (Figure 7k–n). However, during the first 4 h there was an increase in TNF‐α, IL‐1β, and IL‐6. No significant difference was observed in other pro‐inflammatory cytokines like iNOS and IFN‐γ, or anti‐inflammatory cytokines such as IL‐10 and TGF‐β, however a slight increase was observed after 24 h (Figure S20, Supporting Information). To confirm the shift in macrophages polarization toward a restorative phenotype, livers from treated ARE Del^−/−^ mice were stained with anti‐inflammatory YM1^+^ antibodies. This revealed a significant increase of this population in the liver, accompanied by a change in their morphology.

After 4 h from Man‐GNP injection, positive YM1^+^ cells appeared more rounded, indicating an early stage of repolarization of the macrophages (Figure 7o). However after 24 h, YM1^+^ cells exhibited a more elongated shape typical of anti‐inflammatory macrophages (Figure 7p). The quantification of YM1^+^ cells in the hepatic tissue confirmed a significant increase over time (Figure 7q).

To further investigate this repolarization, staining for iNOS^+^ cells, a marker for pro‐inflammatory macrophages, confirmed a significant decrease in the number of these cells in the hepatic tissue (Figure S21, Supporting Information). Furthermore, serum enzymatic levels of sALT, sAST, and sLDH showed a decrease in the number of transaminases in the mice treated and sacrificed after 24 h (Figure 7r, Figure S22, Supporting Information). Because ARE Del^−/−^ mice show damage in the bile ducts, at an advanced stage, we decided to quantify serum bilirubin levels (total and direct) to detect any hepatic decompensation. At 18 weeks, our ARE Del^−/−^ mice had bilirubin levels below the threshold, even in the vehicle, indicating that the mice were still at an early stage of the disease. No significant difference was observed in sALP levels, but also in bilirubin (total and direct) (Figure S22, Supporting Information).

IBA‐1^+^ cells in the liver of mice treated with Man‐GNPs, showed a transient increase in female and an opposite trend in males (Figure S23–S24, Supporting Information). To follow‐up the different hepatic cell populations involved in the pathology, we examined the hepatic immune cell composition of ARE Del^−/−^ mice treated with Man‐GNPs by flow cytometry (Figure 7s–v, Figure S25, Supporting Information). The presence of the sugar had a marked effect on the different hepatic cell populations compared with vehicle mice. The overall percentage of NKP46^+^ cells responsible for the constant production of IFN‐γ in ARE Del^−/−^ mice was reduced (Figure 7s). The presence of Man‐GNPs completely altered the immune milieu and changed all different hepatic cell populations.

The number of B220^+^ cells (Figure 7t), CD3^+^CD4^+^ T cells (Figure 7u), and Ly6C^high^Ly6G^high^ (Figure S25, Supporting Information) was increased in all treated mice compared with the vehicle. Repolarization of macrophages toward an anti‐inflammatory phenotype, led to an increase in IL‐10 secretion which stimulated an increase in B_reg_ cells that simultaneously, induced the development of T_reg_ cells which are CD4^+^T cells.^[^ ^31^ ^]^ Additionally, the percentage of DCs in the liver decreased sharply (Figure S25, Supporting Information), due to a decrease in the production of autoantibodies. Finally, the expression of F4/80^+^CD206^+^ cells, F4/80^+^CD169^+^ cells, and F4/80^+^CD14^+^ cells was examined by flow cytometry. The percentage of F4/80^+^CD206^+^ macrophages (anti‐inflammatory phenotype) increased in the presence of Man‐GNPs, reflecting repolarization of pro‐inflammatory macrophages into a restorative phenotype which was maintained after 24 h of a single administration (Figure 7v). However, F4/80^+^CD169^+^ macrophages in the presence of Man‐GNPs showed initial immunosuppression, but their levels returned to normal after 24 h (Figure S25, Supporting Information). This inhibition of CD169 could be a good prognostic signal, as CD169^+^ macrophages act similarly to DCs. The blocking of this receptor by Man‐GNPs may be linked to the downregulation of DCs and the reduction in autoantibody presentation. Finally, the number of CD14^+^ cells, which are primarily pro‐inflammatory macrophages, decreased as the F4/80^+^CD206^+^ macrophages increased, indicating a repolarization of the macrophages in the liver toward a restorative phenotype (Figure S25, Supporting Information). However, this effect was transient, as the levels returned to normal after 24 h, as previously shown.

### 2.6 In Vitro Repolarization of Macrophages

**Figure 8:**
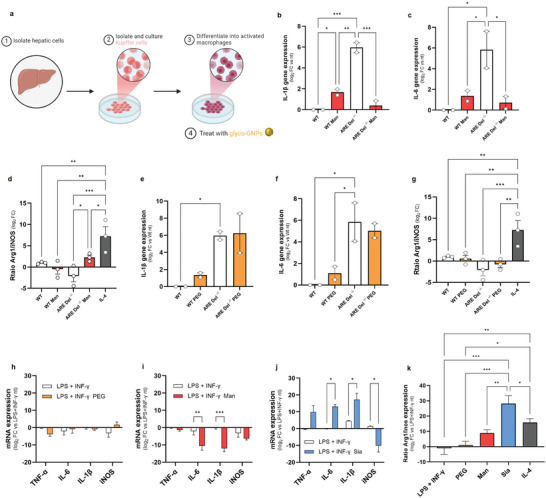
Re‐polarization of hepatic macrophages in vitro. a) Scheme of KCs extraction from ARE Del^−/−^ mice. Image created with Biorender. b–g) Gene expression of hepatic pro‐inflammatory IL‐1β, IL‐6, and Arg1/iNOS ratio in KCs from WT and ARE Del^−/−^ mice treated with b–d) Man‐GNPs and e–g) PEG‐GNPs, and in KCs stimulated with 20 ng mL^−1^ of IL‐4. Data are presented as mean ± SEM of *n* = 2 mice. h–j) mRNA expression of pro‐inflammatory cytokines in BMDMs stimulated with 100 ng mL^−1^ LPS and 20 ng mL^−1^ IFN‐γ and treated with h) PEG‐GNPs, i) Man‐GNPs and j) Sia‐GNPs. Data are presented as mean ± SEM of *n* = 3. k) mRNA expression of anti‐inflammatory cytokines in BMDMs stimulated with LPS and IFN‐γ and treated with PEG‐GNPs, Man‐GNPs and Sia‐GNPs, or with 20 ng mL^−1^ of IL‐4. Data are presented as mean ± SEM of *n* = 3. *p* values were determined by one‐way ANOVA with Bonferroni's correction ^*^ *p* < 0.05, ^**^ *p* < 0.01, ^***^ *p* < 0.001, and ^****^ *p* < 0.0001.

The effect of Man‐GNPs was validated in vitro by isolating KCs from WT and ARE‐Del^−/‐^ mice (**Figure** **8**a–g, Figure S26, Supporting Information). Evaluating the expression of pro‐inflammatory genes, we found that in healthy mice, apart from IL‐1β, there were no significant differences between mice treated with glyco‐GNPs and untreated mice. However, in ARE‐Del^−/−^ mice it shows a high induction of inflammatory genes, treatment with Man‐GNPs for 24 h significantly reduces their expression, indicating a negative effect on the inflammatory phenotype of KCs. This was further supported by the increased expression of the Arg1 gene (an anti‐inflammatory marker), as well as the increased Arg1/iNOS ratio. Stimulation of KCs with PEG‐GNPs did not result in any change of the gene expression in WT ARE‐Del^−/−^ mice, confirming that the inhibitory effect observed with Man‐GNPs on the pro‐inflammatory response is due to the functionalization with the sugar moiety.

This inhibition of Man‐GNPs is not only limited to the inflammatory context of the liver but applies to the nature of inflammatory macrophages. By stimulating in vitro murine bone marrow‐derived macrophages (BMDMs) obtained from WT mice with LPS+IFN‐γ toward a pro‐inflammatory phenotype, BMDMs treated for 24 h with PEG‐GNPs did not show significant modulation (Figure 8h–k, Figure S27, Supporting Information). Man‐GNPs confirmed the inhibition of IL‐6 and IL‐1β genes.

While Sia‐GNPs induced pro‐inflammatory gene expression both in KCs isolated from ARE‐Del^−/−^ mice (Figure S26e–g, Supporting Information) and in BMDMs. The exception is iNOS, which is downregulated after stimulation with Sia‐GNPs in BMDMs activated in a pro‐inflammatory sense, as was highlighted by the increase in the Arg1/iNOS ratio, resulting in an intermediate macrophage phenotype in vitro that should be further explored.

## 3 Discussion

Glucose is an essential nutrient for cell growth, especially for malignant cells that dramatically increase aerobic glycolysis to sustain their rapid proliferation, leading to tumor growth and promoting cell invasion and metastasis.^[^ ^49^, ^50^ ^]^ Also, other cell types such as pro‐inflammatory macrophages, activated DCs, NK cells, effector T cells, and B cells have also been proved to enhance glycolysis.^[^ ^19^ ^]^

Mannose is transported into the cells by the same glucose transporter (GLUT) and then phosphorylated to mannose‐6‐phosphate (M6P) by hexokinases (HK).^[^ ^51^ ^]^ About 95% of M6P is catabolized in glycolysis to form fructose‐6‐phosphate by a mannose phosphate isomerase (MPI). Low MPI levels, cause the accumulation of M6P in the cell, inhibiting the glucose phosphate isomerase (GPI) and other enzymes involved in glucose metabolism, affecting the glucose catalysis and affecting the tricarboxylic acid cycle (TCA), pentose phosphate pathway and glycan synthesis.^[^ ^21^ ^]^

Current therapies targeting CD206 on anti‐inflammatory macrophages with polymeric NPs to deliver doxorubicin to TME^[^ ^52^ ^]^ have demonstrated to be possible to target TAMs. For example, new strategies exploiting modified siRNA expressing Man as a ligand improved its affinity to TAMs.^[^ ^53^ ^]^ Other groups have demonstrated that the presence of Man on NPs increased the amount of macrophages in the target area.^[^ ^54^ ^]^ Coating manganese dioxide NPs with Man and encapsulating hyaluronic acid (HA) promoted the decomposition of hydrogen peroxide in the hypoxic TME transforming TAMs into pro‐inflammatory macrophages.^[^ ^55^ ^]^ Nowadays, most of Man ligand targeting strategies are applied for macrophage repolarization.

On the other hand, Sia has been demonstrated to have a high affinity for receptor Siglec‐1, overexpressed in TAMs.^[^ ^56^ ^]^ Siglec‐1 is very suitable as a targeting target as an endocytosis‐guiding receptor. Nanocarriers using Sia modified amphiphilic egg phosphatidylglycerol structure accumulated at the TAM rich site, enhancing ibrutinib effect and reducing off‐targeting.^[^ ^57^ ^]^ Meanwhile Sia cyclodextrin carriers achieved complex TME in prostate cancer, delivering siRNA to TAMs, causing reprogramming.^[^ ^58^ ^]^ Here we show that using glycans to target specific pattern recognition receptors expressed in macrophages such as CD206 and CD169 is a viable immunotherapy that can be used to treat liver diseases such as CRC hepatic metastases or PBC, two completely opposite pathologies.

Tumor cells must survive to drastic changes in the TME such as hypoxia, nutrient availability, and acidic pH, giving them a remarkable plasticity to adapt to these metabolic changes.^[^ ^3^, ^59^, ^60^ ^]^ Reprogramming glucose metabolism in TAMs can affect the TME, by detecting and eliminating tumor cells.^[^ ^8^ ^]^ However, cancer cells can develop different mechanisms to escape the immune response at genetic, epigenetic, and metabolic levels.^[^ ^22^, ^26^ ^]^ It has been observed that pro‐inflammatory macrophages present a disrupted TCA cycle, leading to the accumulation of citrate and succinate.^[^ ^50^ ^]^ The metabolites are involved in several processes such as the synthesis of fatty acids, generation of nitric oxide, and prostaglandins. The accumulation of citrate and succinate affects different cellular functions. For instance, succinate is involved in the sustained production of IL‐1β through hypoxia‐inducible factor 1‐α (HIF1α)^[^ ^61^ ^]^ leading to a drop in mitochondrial activity and lactate accumulation in the cells.^[^ ^50^ ^]^ The accumulation of lactate can inhibit the activation of T cells and DCs, observed in the case of CD4^+^ T cells, preventing cytokine release and monocyte migration, and upregulating Arg1.

Meanwhile, in ARE Del^−/−^ mice a completely opposite situation occurs. The presence of Man‐GNPs reduces the expression of IL‐1β, leading to the reduction in other pro‐inflammatory cytokines such as TNF‐α and IL‐6. This occurs because there is no accumulation of HIF1α, and it is dependent on IL‐1β. As a result, this changes the behavior of macrophages to a restorative phenotype. Anti‐inflammatory macrophages present an unimpaired TCA cycle and obtain their energy through fatty acid oxidation.^[^ ^50^ ^]^ Fatty acid oxidation has an important role in the downregulation of DCs and regulating T‐cell responses, as promoting the development of T_reg_ cells while inhibiting effector T cells.^[^ ^50^ ^]^ Meanwhile, the sustained expression of IL‐10 over time explains the activation of B cells in the presence of the glyco‐NPs as they stimulate the immunosuppressive phenotype of B_reg_ cells.

This concludes that glycans can be used as anti‐inflammatory drugs in the murine model ARE Del^−/−^ and as an immune therapy for TAMs in the TME. The presence of glycans on the GNPs specifically targets macrophages, allowing them to be repolarized into a restorative phenotype in the liver. Future studies assessing its efficacy in other types of inflammatory diseases or other solid tumor types would be of great interest to generalize the potential of glyco‐GNP therapy.

Overall, these data suggest that both sugars affect macrophages, but only Man can alter their phenotype in two completely opposite situations, supporting the use of glycans for specific targeting strategies that can be used as immunotherapies. However, depending on the type of stimuli, macrophages repolarization and classification into pro‐ or anti‐inflammatory is not arbitrary, as both populations always co‐exist.^[^ ^4^, ^7^, ^8^, ^13^, ^62^, ^63^ ^]^

## 4 Conclusion

Given the lack of immune therapies able to target specific cell types in the liver microenvironment, the display of glycans such as Man or Sia on the NPs surface, promoted the repolarization of macrophages toward a restorative phenotype. Due to the chronic nature of both pathologies, hepatic metastasis and PBC, and the lack of treatments, we developed nanodevices that specifically target activated macrophages within the liver. Altogether, our results show the potential of glycans as specific‐targeting molecules, highlighting the importance of mimicking nature and gaining more insight into these activation‐dependent mechanisms. Our findings validate this nanotherapeutic approach and confirms macrophages repolarization as a druggable target. However, further studies on the mechanisms of this repolarization must be considered. Exposing mice to longer periods and repetitive administrations is necessary to understand if consecutive exposure to the glyco‐NPs may activate other mechanisms in the immune cells leading to the development of secondary effects such as hepatic toxicity or immunity resistance toward the presence of glycans.

This is the first step demonstrating the feasibility of using glycans as sensitive‐targeting molecules in GNPs and especially, the potential of Man to be used in clinical applications for remission of both diseases.

## 5 Experimental Section

### Materials

Before use, all glassware was washed with aqua Regia to remove metallic particles and rinsed thoroughly with Milli‐Q water. All chemicals were used as received. Hydrogen tetrachloroaurate (III) hydrate (HAuCl_4_), ascorbic acid (AA), pentafluorophenol (PFP), 11‐[(methylcarbonylthio)undecyl]tri(ethylene glycol) acetic acid (EG_6_C_11_SH), ethyl‐3‐(3‐dimethyl aminopropyl)carbodiimide (EDC), *N*,*N*‐dimethylformamide (DMF), hydrochloridric acid, nitric acid, dimethyl chloride (DMC), *N*,*N*‐diisopropylethylamine (DIPEA), methanol, sodium methoxide, tris base (99%), acrylamide/bis‐acrylamide 40% solution, sodium dodecyl sulfate (SDS), ammonium persulphate, *N*,*N*,*N*,*N*‐tetramethylethylenediamine (TEMED), phosphate buffer saline (PBS) tablets, and D‐(+)‐Sucrose were purchased from Sigma‐Aldrich. Imperial Protein Stain and Pierce C18 Tips were purchased from Thermo Scientific Ireland. 3× Blue Loading Buffer and 30× Reducing Agent (1.25 M DTT) were purchased from Cell Signaling Technology. LPS from *Escherichia coli* serotype 055:B5 was purchased from Sigma‐Aldrich.

### Analytical Methods

Reactions were monitored by thin‐layer chromatography (TLC) on Silica Gel 60 F254 (Sigma Aldrich). Compounds were visualized by heating with 10% (v/v) ethanolic H_2_SO_4_ and 20% (v/v) methanolic H_2_SO_4_. Column chromatography was performed using Silica Gel 200–400 mesh.

^1^H NMR spectra were measured at 400 MHz and 298 k with a Bruker Avance III spectrometer; *δ*_H_ values were reported in ppm, relative to the internal standard (*δ*_H_ = 3.31, CD_3_OD). ^13^C NMR spectra were measured at 100 MHz and 298 k with a Bruker AvanceIII spectrometer; *δ*_c_ values are reported in ppm relative to the signal of CD_3_OD (δ_C_ = 49.0, CD_3_OD). NMR signals were assigned by homonuclear and heteronuclear 2‐dimensional correlation spectroscopy (COSY, HSQC).

### Synthetic Procedures—Synthesis of PFP‐EG_6_C_11_SH (1)

11‐[(methylcarbonylthio)undecyl]tri(ethylene glycol) acetic acid (70 mg, 0.16 mmol) and EDC (51 mg, 0.33 mmol) were suspended in dry DMF (7 mL) and treated with PFP (60 mg, 0.33 mmol). The suspension was left at RT and stirred for ≈18 h. The crude was concentrated under reduced pressure and further purified by flash column chromatography (hexane‐ethyl acetate 7:3 → ethyl acetate 100%) obtaining **1** as a brown oil (17.7 mg, 42% purity) but with some traces of unreacted PFP.

^1^H NMR (400 MHz, methanol‐*d_4_*): *δ*_H_ (ppm) = 4.65 (s, 2H, OCH_2_COO), 3.82 (t, 2H, OCH_2_CH_2_O), 3.75 (t, 2H, OCH_2_CH_2_O), 3.67 (t, 2H, OCH_2_CH_2_O), 3.65 (t, 2H, OCH_2_CH_2_O), 3.59 (t, 2H, OCH_2_CH_2_O), 3.5 (t, 2H, OCH_2_CH_2_O), 2.9 (t, 2H, OC**H_2_** (CH_2_)_9_CH_2_S)), 2.82 (t, 2H, OCH_2_ (CH_2_)_9_C**H_2_** S), 2.34 (s, 3H, CH_3_), 1.56 (m, 2H, OCH_2_C**H_2_** (CH_2_)_8_CH_2_S), 1.35 (m, 16H, OCH_2_(C**H_2_**)_9_CH_2_S).

^19^F NMR (400 MHz, methanol‐*d_4_*): *δ*_F_ (ppm) = ‐155 (m, 2F, aromatic C‐F), ‐161 (m, 1F, aromatic C‐F), ‐166 (m, 2F, aromatic C‐F).

### Synthetic Procedures—Glycosylation with 3‐Aminopropyl‐*N*‐Acetylneuraminic Acid (**2**)

Compound **1** (8.7 mg, 0.014 mmol) and DIPEA (9 µL, 0.056 mmol) were dissolved in dry DMC (800 µL) and treated with amino‐sialic acid (8.5 mg, 0.025 mmol) dissolved in dry DMF (400 µL, 8.5 mg, 0.025 mmol) dropwise added. The reaction was left stirring at RT for 36 h. The crude was concentrated under reduced pressure, dissolved in water, and purified by a G10 Sephadex column. Compound 2 (20.2 mg, quantitative) was dialyzed and freeze‐dried, confirming the successful attachment of compound **1** with the amino group of the sialic acid by TLC and ^1^H NMR.

^1^H NMR (400 MHz, methanol‐*d_4_*): *δ*_H_ (ppm) = 3.92 (t, 2H, sialic acid), 3.75 (m, 2H, OCH_2_CH_2_O), 3.58 (m, 6H, OCH_2_CH_2_O), 3.51 (m, 2H, OCH_2_CH_2_O), 3.40 (m, 2H, OCH_2_CH_2_O), 2.78 (m, 4H, OC**H_2_** (CH_2_)_9_C**H_2_** S), 2.2 (t, 3H, CH_3_), 1.93 (m, 2H, sialic acid), 1.65 (m, 3H, CH_3_), 1.49 (m, 2H, sialic acid), 1.22 (m, 18H, OC**H_2_** (C**H_2_**)_9_CH_2_S).

### Synthetic Procedures—Removal of Protecting Group SH (3)

Compound **2** (20.2 mg, 25.7 mmol) was dissolved in MeOH (4 mL) and treated with MeONa (31 g, 0.36 mmol) to reach pH 9 and was left stirring at RT for 1 h. The reaction was quenched with amberlite resin still has a neutral pH. The resin was filtered and compound 3 (34.9 mg, quantitative) was concentrated under reduced pressure without any further purification.

### NP Synthesis, Functionalization, and Characterization—Synthesis of Spherical Gold Nanoparticles (GNPs)

Spherical GNPs were synthesized in a bench‐top reactor, operating in a microfluidic flow regime, as reported by Polito et al.^[^ ^29^ ^]^ The apparatus comprises a dual syringe pump (Legato 200, KD Scientific) working at 5 mL min^−1^ flow rate, connected through a PEEK T‐mixer to a PTFE tube reaction coil (ID 1 mm, length 160 mm). In a typical procedure, syringe (A) is loaded with a solution of L‐AA (20 mL, 0.8 × 10^−3^ m) and the other one (B) is loaded with a solution containing HAuCl_4_ (20 mL, 0.2 × 10^−3^ m). The colloidal solution was collected and immediately treated with a solution with of PEG or glycans to quench gold growth.

### NP Synthesis, Functionalization, and Characterization—GNP Functionalization

A stock solution of the amphiphilic linker, EG_6_C_11_SH, with a total concentration of 2 mg mL^−1^ was prepared in Milli‐Q water and added the solution of 30 nm particles (200 mL). The solution was left stirring at RT for 48 h (NP1). The monosaccharides, mannose and sialic acid, already attached to the amphiphilic linker were diluted in Milli‐Q water containing a glycan concentration of 4 mg mL^−1^ and added to the 30 nm particles solution (200 mL). The solutions were left stirring at RT for 48 h (NP2; NP3). All spherical GNPs were then purified by centrifugation at 6000 rpm to remove residual AA and/or unreacted precursor. The particles (NP1, NP2, and NP3) were re‐suspended in water and characterized by UV–Vis, DLS, and TEM.

### NP Synthesis, Functionalization, and Characterization—GNP Characterization

UV–Vis absorption spectra of the NPs were acquired across a range of wavelengths 400–1000 nm with UV–Vis spectroscopy (Agilent 8453) to study the colloidal stability of NPs after their surface modifications. Measurements were carried out in Milli‐Q water with a cuvette path length of l path = 1 cm. NPs concentration was determined by ICP‐MS measurement to determine the mass of gold, which was converted into the total number of NPs dispersed in the solution. Note that it was assumed that the surface coating does not have any influence.

Transmission electron microscope (TEM) (Zeiss LIBRA 200FE, equipped with: 200 kV FEG, in column second‐generation omega filter for energy selective spectroscopy (EELS) and imaging (ESI), HAADF STEM facility, EDS probe for chemical analysis, integrated tomographic HW and SW system) was used to characterize the quality of glyco‐GNPs. Samples for TEM analysis were prepared by depositing a drop (4 µL) of diluted NPs solution on top of a carbon‐coated copper grid (300 mesh) and left to dry at RT. The particle size distribution was obtained by measuring at least 100 NPs.

The hydrodynamic diameter (*d*_h_) and zeta potential (*ζ*‐potential) of a suspension of glyco‐GNPs (1 mL) were measured by dynamic light scattering (DLS), using a 90 Plus Particle Size Analyzer (Brookhaven Instrument Corporation) operating at 15 mW of a solid‐state laser (*λ* = 661 nm), using a scattering angle of 90 °C. All samples were equilibrated for 5 min at ≈25 °C to ensure NP motion was due to Brownian motion and not due to any thermal gradients. The determined *d*_h_ (nm) and *ζ*‐potentials (mV) were the averages of at least three independent measurements.

### Animal Experiments

The Institute for Pharmacological Research Mario Negri IRCCS adheres to the principles set out in the following laws, regulations, and policies governing the care and use of laboratory animals: Italian Governing Law (D.lgs 26/2014; Authorization n.19/2008‐A issued March 6, 2008 by Ministry of Health); Mario Negri Institutional Regulations and Policies providing internal authorization for people conducting animal experiments (Quality Management System Certificate‐UNI EN ISO 9001:2015‐Reg. N°6121); the NIH Guide for the Care and Use of Laboratory Animals and EU directives and guidelines (EEC Council Directive 2010/63/UE). Animal studies in healthy and ARE Del^−/−^ mice were approved by the Mario Negri Institute Animal Care and Use Committee (IACUC) and by the Italian National Institute of Health (code no. 49/2021‐PR and 58/2020‐PR). Animal experiments with murine cancer models were approved by the Animal Care and Use Committee of the San Raffaele Scientific Institute (SRSI, IACUC, 808 and 1042). All animals were kept under specific pathogen‐free (SPF) conditions in the Institute's animal care facilities. Animals were bred in rooms with a constant temperature of 21 ± 1 °C, humidity of 55 ± 10%, a 12‐h light‐dark cycle, and ad libitum access to food and water. All mice were regularly checked by a veterinarian responsible for monitoring animal welfare and reviewing experimental protocols.

### In Vivo Safety Study

Female, 18‐week‐old CD1 mice (Charles River, Italy) were injected intravenously with GNPs at a dose of 2 × 10^11^ NPs per mice diluted in 200 µL of injection‐grade distilled water (54.63 µg of GNPs per mouse) and euthanized 4 and 24 h later. Mice were anesthetized and serum was collected from retro‐orbital bleeding to analyze toxicity markers. Liver samples were used for histology and flow cytometry.

### Mouse Model of Liver Metastases

A total of 8‐to 10‐week‐old age‐matched immune competent male C57B/6J mice (Charles River Laboratory, Calco, Italy) were injected with 7 × 10^4^ MC38 (H‐2b, C57BL/6‐derived) a syngeneic CRC cell line, through the superior mesenteric vein as previously described.^[^ ^43^, ^64^ ^]^ For superior mesenteric vein injections, deep anesthesia was induced by isoflurane inhalation (5% induction and 2% for maintenance in 2 L min^−1^ oxygen). CRC cells were injected using a 29G needle and to prevent excessive bleeding, vein puncture was compressed with a sterile and absorbable hemostatic gauze (TABOTAMP). The peritoneum and skin were sutured with silk 4.0 and 7 mm wound clips as described.^[^ ^43^, ^64^ ^]^ The MC38 CRC cell line was routinely passaged in vivo to establish a liver metastases‐prone cell line and cultured in vitro under standard conditions at 37 °C in a humid atmosphere with 5% CO_2_ in DMEM GlutaMAX medium (Gibco) supplemented with FBS (10%) (Lonza) and P/S (1%) (Gibco). All cells were routinely tested for mycoplasma contamination using the N‐GARDE Mycoplasma PCR reagent set (EuroClone). Cell number and dimension were routinely assessed by an automated CytoSMART cell counter (Corning), and cell identity was confirmed by morphology. Twenty‐one days after MC38 injection, liver metastases were identified as focal lesions showing slight hyper‐intensity on T2‐weighted images and concurrent hypo‐intensity on contrast‐enhanced HBP T1‐weighted images using MRI performed at the Experimental Imaging Center of SRSI on a preclinical 7‐Tesla MR scanner (Bruker, BioSpec 70/30 USR, Paravision 6.0.1, Germany) equipped with 450/675 mT/m gradients (slew rate: 3400/4500 T/m s^−1^; rise time 140 µs), coupled with a dedicated four channels volumetric mouse body coil. Gadoxetic acid (Gd‐EOB‐DTPA) was intravenously injected, at a dose of 0.05 µmol g^−1^ of body weight, for contrast enhancement as previously described.^[^ ^43^, ^64^, ^65^ ^]^ MRI images were analyzed by using Mipav software (5.3.4 and later versions, Biomedical Imaging Research Services Section, ISL, CIT, National Institute of Health, USA).

### Mouse Model of Primary Biliary Cholangitis (PBC)

The generation of ARE‐Del^−/−^ mice, as well as the PBC phenotype has been previously described.^[^ ^66^ ^]^ ARE Del^−/−^ mouse model is characterized by a 162 nt ARE region deletion in the 3′ untranslated region (3′UTR) of the interferon‐gamma (IFN‐γ) gene that results in chronic circulating serum IFN‐γ levels. Homozygous ARE‐Del^−/−^ mice present both serologic and cellular abnormalities typical of patients with PBC.

### Use of GNPs in Mouse Models of Liver Diseases

To define the effect of glyco‐NPs in mouse models of liver disease characterized by different polarized intrahepatic macrophages, 18‐week‐old, female and male C57BL/6 ARE Del^−/−^ (Charles River, Italy) and 12‐week‐old C57BL/6 mice harboring MC38 liver metastatic tumors were intravenously injected with GNPs at a dose of 2 × 10^11^ NPs per mice diluted in 200 µL of injection grade distilled water. An additional group of mice was not injected with CRC cells serving as Sham control. Four hours and 24 h later, mice were anesthetized and blood collected from retro‐orbital plexus, and serum were analyzed for markers of cellular toxicity. Groups of mice were euthanized at 4 and 24 h and livers were collected in 10% neutral‐buffered formalin for histological analysis or processed for the isolation of liver nonparenchymal cells (NPCs) for flow cytometry, as previously described.^[^ ^43^, ^64^ ^]^

### Serum Biochemical Analysis

Markers of cell toxicity such as serum aspartate aminotransferase activity (sAST) or hepatocellular injury/toxicity such as serum alanine aminotransferase activity (sALT), lactate dehydrogenase (sLDH), and alkaline phosphatase (sALP) were monitored after GNPs administration and expressed as U L^−1^. To evaluate liver functionality in the ARE Del^−/−^ mouse model, total serum bilirubin and direct bilirubin levels were measured and expressed as mg dL^−1^. All enzyme activities were evaluated using the kinetic UV method optimized by the IFCC (International Federation of Clinical Chemistry and Laboratory Medicine) in an Aries chemical analyzer (Werfen Instrumentation Laboratory S.p.A., Italy). Each analysis was validated by a biochemical chemist and hematologist using quality control serums (CQI), in the San Raffaele Mouse Clinic.

### Isolation of Liver Nonparenchymal Cells (NPCs) and Kupffer Cells (KCs)

Liver NPCs, including leukocytes and KCs, were isolated from control or GNP‐treated mice at 4 and 24 h after injection. The 4‐h time point was chosen because GNPs of similar characteristics have shown full accumulation in target organs while 24 h represent a specific time point in which GNPs could shape target cell immune phenotype.^[^ ^32^, ^67^ ^]^ Briefly, after euthanasia, livers were perfused through the inferior vena cava with PBS (10 mL) to remove circulating blood cells. Livers were cut into small pieces and incubated for 15 min at 37 °C in digestion medium (10 mL) (RPMI GlutaMAX medium[Gibco] containing 200 U mL^−1^ of collagenase type IV[Sigma‐Aldrich] and 100 U mL^−1^ of DNAse I [Sigma‐Aldrich]). The remaining undigested fragments were passed through an 18G needle using a syringe and filtered through a 70 µm cell strainer to obtain a single cell suspension. Cells were centrifuged for 6 min at 400 rpm at 4 °C and the pellet containing hepatocytes was discarded. The resulting cell suspension of NPCs was incubated with ACK lysis buffer (Lonza) for 30 s to remove red blood cells, and washed with cold RPMI. NPCs were counted and processed for flow cytometry analysis.^[^ ^43^, ^64^ ^]^

For KCs isolation, the resulting cell suspension of NPCs was placed into a 6‐well plate at a density of 1–3 × 10^7^ cells per well in complete Dulbecco's Modified Eagle's Medium (DMEM) and incubated for 2 h in a 5% CO_2_ atmosphere at 37 °C. Nonadherent cells were then removed from the dish by gently washing with PBS, leaving the adherent cells, which were KCs. They were further cultured and used for experiments in complete DMEM supplemented with 50 ng mL^−1^ of recombinant M‐CSF.^[^ ^68^ ^]^

### Flow Cytometry Analysis

Cells were resuspended in PBS and Viakrome 808 Fixable viability dye (Beckman Coulter) and incubated for 10 min at RT in the dark to determine cell viability. Cells were then blocked with FACS buffer (PBS supplemented with 2% FBS) and stained for surface markers using the following antibodies: anti‐CD45R/B220 (clone RA3‐6B2, Biolegend), anti‐CD11b (clone M1/70, Biolegend), anti‐ CD11c (clone N418, Biolegend), anti‐CD3 (clone 145‐2C11, Thermo Fisher Scientific), anti‐CD45 (clone 30‐F11, BD Biosciences), anti‐F4/80 (clone BM8, Biolegend), anti‐CD206 (clone C068C2, Biolegend), anti‐CD169 (clone 3D6.112, Biolegend), anti‐CD4 (clone RM4‐5, Biolegend), anti‐Ly6G (clone 1A8, Biolegend), anti‐Ly6C (clone HK1.4, Biolegend), anti‐NKP46 (clone 29A1.4, Biolegend), anti‐CD31 (clone MEC13.3, BD Biosciences), and anti‐CD14 (clone Sa14‐2, BD Biosciences) for 20 min at 4 °C. Single cell suspensions were subsequently analyzed by flow cytometry with Cytoflex LX instrument (Beckman coulter), and the data were analyzed using Kaluza software (Beckman Coulter).

### RNA Extraction and rt‐qPCR

RNA was extracted from the liver using TRIzol reagent (Thermo Fischer Scientific) according to the manufacturer's instructions. For real‐time qPCR analysis, total RNA (1 µg) was reverse transcribed into complementary DNA using the high‐capacity cDNA reverse transcription kit (Applied Biosystems). The cDNAs were mixed with Quantifast SYBR Green Master Mix (BiotechRabbit) according to the manufacturer's instructions and with both forward and reverse primers (8 pmol) for detecting mRNA levels of the following mouse genes: TNF‐α, IL‐1β, IL‐6, iNOS, Arg1, IL‐10, and TGF‐β (Table S1, Supporting Information). qPCR was carried out using a 7900 fast real‐time PCR system (Applied Biosystems) and the amplification steps were 50 °C for 2 min, 95 °C for 10 min followed by 40 cycles of 95 °C for 15 s and 60 °C for 1 min. The relative quantification of each mRNA was carried out with the comparative threshold cycle method using β‐actin for the normalization of in vivo studies and expressed as fold change compared to untreated control.

### Immunohistochemistry and Autometallography (AMG)

At the time of autopsy for each mouse, livers were sampled, fixed in 10% neutral buffered formalin (Bio‐Optica, Italy) for at least 24 h at RT and then were processed for paraffin embedding. Tissue micrometric sections (4 µm in thickness) were cut with Leica RM55 microtome (Leica Microsystem, Italy) and dried in the oven at 37 °C overnight. To visualize the presence of gold agglomerates in the liver parenchyma, autometallography (AMG) staining was carried out, as previously described.^[^ ^67^ ^]^ Hematoxylin and eosin (H&E) staining was performed in liver sections treated with glyco‐GNPs and vehicle‐treated mice. Hematoxylin stains cell nuclei blue, and eosin stains the extracellular matrix and cytoplasm pink. Nuclei were stained with Mayer's hematoxylin solution (Bio‐Optica, Italy) for 2 min and 30 s, and then washed with water till they came in color. The counterstain was carried out in eosin Y solution (Bio‐Optica, Italy) for 1 min and 20 s to stain the cytoplasm. The slides were then washed in tap water until discoloration occurred. Specimens were dehydrated and embedded with a xylene‐based mounting medium (DPX, Sigma). IBA‐1 staining was performed in liver sections treated with GNPs and vehicle‐treated mice. IBA1 stains macrophages brown and hematoxylin stains nuclei blue. HIER was performed with citrate buffer pH = 6 for 30 min at 95 °C, inhibition of endogenous peroxidase with H_2_O_2_ 3% for 10 min at RT, and incubation with blocking solution (PBS‐NGS 10%‐Tween 20 0.05%) for 30 min at RT. For subcellular localization, IBA‐1 (1:200, Wako Chemicals, USA) was used to label the macrophage calcium‐binding protein, followed by an amplification step with the labeling system ABC (Vectastain Elite) and the chromogenic reaction with DAB (Sigma‐Aldrich). Cell nuclei were stained with Mayer's hematoxylin solution (Bio‐Optica, Italy) for 30 s and then washed with water until discolored. Samples were dehydrated and fixed with a xylene‐based mounting medium (DPX, Sigma). Sirius red staining was performed in liver sections from ARE Del^−/−^ mice treated with glyco‐GNPs and sham mice. Sirius red‐stained most of the tissue yellow and in red the collagen fibers surrounding the hepatic veins and sinusoids. Collagen fibers were stained with Picro‐Sirius Red solution (Bio‐Optica, Italy) for 1 h and then washed with acidified water. The samples were dehydrated with an alcohol scale. Glasses were dried under a fume hood and mounted with a xylene‐based mounting medium (DPX, Sigma). All images were acquired using Olympus BX61VS.

### Hard Corona (HC) Obtention

In order to study the extrinsic physicochemical and biomolecular characteristics of the different GNPs, different approximations has to be performed to mimic real bloodstream conditions. It is assumed that plasma represents 80% of the total interacting volume. Given that there are 2 × 10^11^ NPs per mouse and each GNP has a nominal diameter of 30 nm, the mass of GNP per mouse is 54.63 µg. By dividing the total GNP mass by total plasma volume, the concentration of GNPs in healthy mice plasma is assumed to be 33.35 µg mL^−1^. PBS (400 µL) was introduced to low protein binding 1.5 mL microtubes. Glyco‐GNPs were lastly introduced to the microtubes to reach a final concentration of 33.35 µg mL^−1^. The total sample volume was 500 µL. Then, the solution was incubated at 37 °C for 1 h under continuous agitation at 300 rpm. After incubation, the NP corona complex was pelleted from excess proteins by centrifugation at 7500 g, 4 °C for 15 min. The supernatant was discarded, and the pellet was then resuspended in PBS (500 µL) and centrifuged again. The washing procedure removes unbound and loosely bound proteins from the NPs. The NP‐HC complexes are obtained after repeating the washing procedure three times.

### Differential Centrifugal Sedimentation (DCS)

Differential centrifugal sedimentation experiments were performed using a CPS Disc Centrifuge DC24000 (Analytik Ltd.), using the standard sucrose gradient of 8−24%. PVC NPs (0.559 µm size) were used as calibration standards for each sample measurement. The time it takes for spherical particles with uniform density to travel from the center of the disk to the detector is directly related to their size. However, for particles that are heterogeneous or irregularly shaped, variations in arrival times still enable differentiation between populations, although their sizes should be regarded as “apparent” sizes.^[^ ^69^, ^70^ ^]^ As the protein layer is less dense than the GNPs, the NP–corona complex has a lower density, and the arrival time to the detector is delayed. This leads to a different NP apparent size, which can be directly related to the protein shell thickness using the core−shell model.^[^ ^71^ ^]^

### SDS‐PAGE

SDS‐PAGE analysis of the biomolecular corona was carried out following protocols optimized in lab.^[^ ^72^ ^]^ The NP‐HC was resuspended after the last centrifugation step with PBS (12 mL). 3× Blue Loading Buffer (6 mL) and 30× Reducing Agent (ratio 10:1) to reach the final buffer composition and heated at 95 °C for 5 min. The samples were then loaded in a 12% polyacrylamide gel freshly made in the lab. Gel electrophoresis was performed at 120 V, 400 mA for about 90 min each, until the proteins neared the end of the gel. The gels were stained using Blue Coomassie, following the manufacturer's instructions.

### Mass Spectrometry (MS)

To determine the protein composition of the HC complexes, the GNP‐HC samples were run by SDS‐PAGE and gel bands were cut out following the in‐gel trypsin digestion protocol as previously described.^[^ ^72^ ^]^ Digested peptides were then resuspended in 0.1% formic acid (40 µL). Then the peptides (500–1000 ng) were loaded onto Evotips as per the manufacturer's instructions (EvoSep). Briefly, Evotips were activated by soaking them in isopropanol, primed with buffer B (ACN, 0.1% FA, 20 µL) by centrifugation for 1 min at 700 g. Tips were soaked in isopropanol and equilibrated with buffer A (MS grade water, 0.1% FA, 20 µL) by centrifugation. Another buffer A (20 µL) was loaded onto the tips and the samples were added on top of that. Tips were centrifuged and washed with buffer A (20 µL) followed by overlaying the C18 material in the tips with buffer A (100 µL) and a short 20 s spin.

The samples were analyzed by the Mass Spectrometry Resource (MSR) in University College Dublin on a Bruker TimsTOF Pro mass spectrometer connected to a Evosep One chromatography system. Peptides were separated on an 8 cm analytical C18 column (Evosep, 3 µm beads, 100 µm ID) using the pre‐set 33 samples per day gradient on the Evosep one. The Bruker TimsTOF Pro mass spectrometer was operated in positive ion polarity with TIMS (Trapped Ion Mobility Spectrometry) and PASEF (Parallel Accumulation Serial Fragmentation) modes enabled. The accumulation and ramp times for the TIMS were both set to 100 ms, with an ion mobility (1/k0) range from 0.62 to 1.46 Vs cm^−1^. Spectra were recorded in the mass range from 100 to 1700 m/z. The precursor (MS) intensity threshold was set to 2500 and the precursor target intensity was set to 20 000. Each PASEF cycle consisted of one MS ramp for precursor detection followed by 10 PASEF MS/MS ramps, with a total cycle time of 1.16 s.

Bruker mass spectrometric data from the TimsTOF was processed using the MaxQuant (version 2.0.3.0) incorporating the Andromeda search engine. To identify peptides and proteins, MS/MS spectra were matched against a Uniprot Mus musculus (Mouse) database containing 54910 entries (proteome ID UP000000589). All searches were performed using the default setting of MaxQuant, using the unspecific enzyme setting and a false discovery rate of 1% on the peptide and protein level. The database searches were performed with acetylation (protein N terminus) and oxidation (M) as variable modifications. For the generation of label‐free quantitative (LFQ) ion intensities for protein profiles, signals of corresponding peptides in different nano‐HPLC MS/MS runs were matched by MaxQuant in a maximum time window of 1 min. Perseus statistical software (version 2.0.3.0) was used to analyze the LFQ intensities, and data was log‐transformed.

### Bone Marrow‐Derived Macrophages (BMDMs) Preparation

BMDMs were obtained from 18‐week‐old mice from WT mice. The femurs were removed, the attached muscles were cleaned, and the bones were washed in sterile RPMI 1640 medium. The epiphyses of the bones were then cut off, and the bone marrow was flushed out using a syringe filled with RPMI 1640 into a 15 mL sterile polypropylene tube. The collected BMDMs were cultured in complete IMDM medium supplemented with 10% fetal bovine serum (FBS; Lonza), 1% penicillin‐streptomycin (Lonza), 1% glutamine (Lonza), and 20 ng mL^−1^ macrophage colony‐stimulating factor (mM‐CSF). After 7 days of culture, the adherent macrophages (BMDMs) were recovered and used for experiments.

### Statistical Analysis

All tests for physicochemical characterization of NPs were performed at least in triplicate. For in vivo experiments, the number of animals euthanized at each data point was minimized (*n* = 3) according to the 3Rs principle. Data in bar graphs are expressed as mean ± SEM. Statistical analysis was performed using GraphPad Prism (version 10.1.2). In general, two‐way Student's *t*‐test was used when comparing two groups and one‐way ANOVA followed by Bonferroni post hoc analysis when comparing three or more groups. No collected data was excluded from the analysis. The *p*‐value is either reported in the manuscript text or shown in Figures and legends as follows: ^*^ *p* < 0.05, ^**^ *p* < 0.01, ^***^ *p* < 0.001, or ^****^ *p* < 0.0001.

## Conflict of Interest

The authors declare no conflict of interest.

## Author Contributions

J.F.A., P.B., and G.S., conceived the study, interpreted the data and wrote the manuscript; J.F.A., P.P.S., L.P., R.F.M., L.L., G.O., and L.P. synthesized and characterized the glycans and glyco‐GNPs.; J.F.A., N.P., and C.G. performed flow cytometry; J.F.A., F.C., M.B.V., C.B.E., A.M., G.Y.M., and G.S. conducted the in vivo experiments; J.F.A, A.V., and C.F. quantified glyco‐GNPs by ICP‐MS; N.G.S. and M.B.V. did in vitro studies; A.C. and F.F. analyzed samples by TEM; A.M. and M.P.M. evaluated the protein corona studies; J.F.A. and J.K. performed confocal studies; J.F.A. and S.F. conducted rt‐PCR.

## Supporting information



Supporting Information

## Data Availability

The data that support the findings of this study are available in the supplementary material of this article.
