# Supplementary material for: Functional Polarization of Liver Macrophages by Glyco Gold Nanoparticles
Source: Adv Sci (Weinh). 2025 Feb 14;12(16):2407458. doi: 10.1002/advs.202407458 (PMC12021048; doi:10.1002/advs.202407458)
Supplement: Supplementary file 1 — Supporting Information [file ADVS-12-2407458-s001.pdf]

ADVANCED SCIENCE
Open Access

Supporting Information

for *Adv. Sci.*, DOI 10.1002/advs.202407458

Functional Polarization of Liver Macrophages by Glyco Gold Nanoparticles

Jennifer Fernandez Alarcon, Patricia Perez Schmidt, Nicolo Panini, Francesca Caruso, Martina B. Violatto, Naths Grazia Sukubo, Alberto Martinez-Serra, Charlotte Blanche Ekalle-Soppo, Annalisa Morelli, Giulia Yuri Moscatiello, Chiara Grasselli, Alessandro Corbelli, Fabio Fiordaliso, Joe Kelk, Laura Petrosilli, Giuseppe d'Orazio, Ruth Mateu Ferrando, Ariadna Verdaguer Ferrer, Cristina Fornaguera, Luigi Lay, Stefano Fumagalli, Sandro Recchia, Marco P. Monopoli, Laura Polito, Paolo Bigini* and Giovanni Sitia**

Functional Polarization of Liver Macrophages by Glyco Gold Nanoparticles

Jennifer Fernandez Alarcon[1,2]*, *Patricia Perez Schmidt*[3], *Nicolo Panini*[4], *Francesca Caruso*[5], *Martina B. Violatto*[1], *Naths Grazia Sukubo*[11], *Alberto Martinez-Serra*[6], *Charlotte Blanche Ekalle-Soppo*[5], *Annalisa Morelli*[1], *Giulia Yuri Moscatiello*[1], *Chiara Grasselli*[4], *Alessandro Corbelli*[1], *Fabio Fiordaliso*[1], *Joe Kelk*[7], *Laura Petrosilli*[8], *Giuseppe d'Orazio*[8], *Ruth Mateu Ferrando*[8], *Ariadna Verdaguer Ferrer*[9], *Cristina Fornaguera*[2], *Luigi Lay*[8], *Stefano Fumagalli*[7], *Sandro Recchia*[10], *Marco P. Monopoli*[6], *Laura Polito*[2,12], *Paolo Bigini*[1,12]*, *and Giovanni Sitia*[4,12]*

[1] Department of Molecular Biochemistry and Pharmacology, Istituto di Ricerche Farmacologiche Mario Negri IRCCS, Via Mario Negri 2, 20156 Milano, Italy.

[2] Grup d'Enginyeria de Materials (GEMAT), Institut Químic de Sarrià (IQS), Universitat Ramon Llull (URL), Via Augusta 390, 08017, Barcelona, Spain.

[3] Istituto di Scienze e Tecnologie Chimiche "Giulio Natta", SCITEC-CNR, Via G. Fantoli 16/15, 20138 Milano, Italy

[4] Department of Oncology, Istituto di Ricerche Farmacologiche Mario Negri IRCCS, Via Mario Negri 2, 20156 Milano, Italy.

[5] Experimental Hepatology Unit, Division of Immunology, Transplantation and Infectious Diseases, IRCCS San Raffaele Scientific Institute, Via Olgettina 58, 20132 Milano, Italy.

[6] Department of Chemistry, Royal College of Surgeons of Ireland RCSI, St Stephens Green 123, Dublin, Ireland

[7] Department of Neurosciences, Istituto di Ricerche Farmacologiche Mario Negri IRCCS, Via Mario Negri 2, 20156 Milano, Italy.

[8]Department of Organic Chemistry, University degli Studi di Milano, Via Golgi 19, 20133 Milano, Italy.

[9] Department of Analytical and Applied Chemistry, Institut Químic de Sarrià (IQS), Universitat Ramon Llull (URL), Via Augusta 390, 08017, Barcelona, Spain.

[10] Department of Science and High Technology, University of Insubria, Via Valleggio 11, 22100 Como, Italy.

[11] School of Medicine and Surgery, University of Milano-Bicocca, Piazza dell'Ateneo Nuovo, 1, 20126 Milano, Italy.
[12]Co-last authors

*** Corresponding authors: jennifer.fernandez@iqs.url.edu (Jennifer Fernandez Alarcon), sitia.giovanni@hsr.it (Giovanni Sitia) and paolo.bigini@marionegri.it (Paolo Bigini)**

Keywords: *gold nanoparticles, glycans, immunotherapy, hepatic metastases, primary biliary cholangitis*

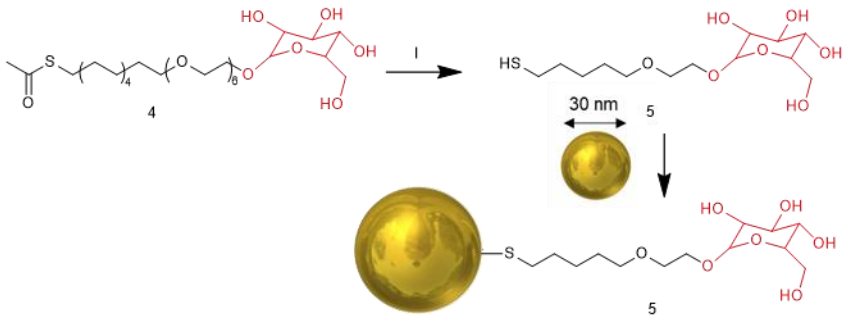

Figure S1 | Synthetic scheme of mannose GNPs. **I)** 29-Thio[3,6,9,12,15,18-hexaoxaundecanyl-α-D-mannopyranoside] was reacted with MeOH/MeONa, pH = 9, RT, 1 hour. This reaction has been already detailed described in literature[1].

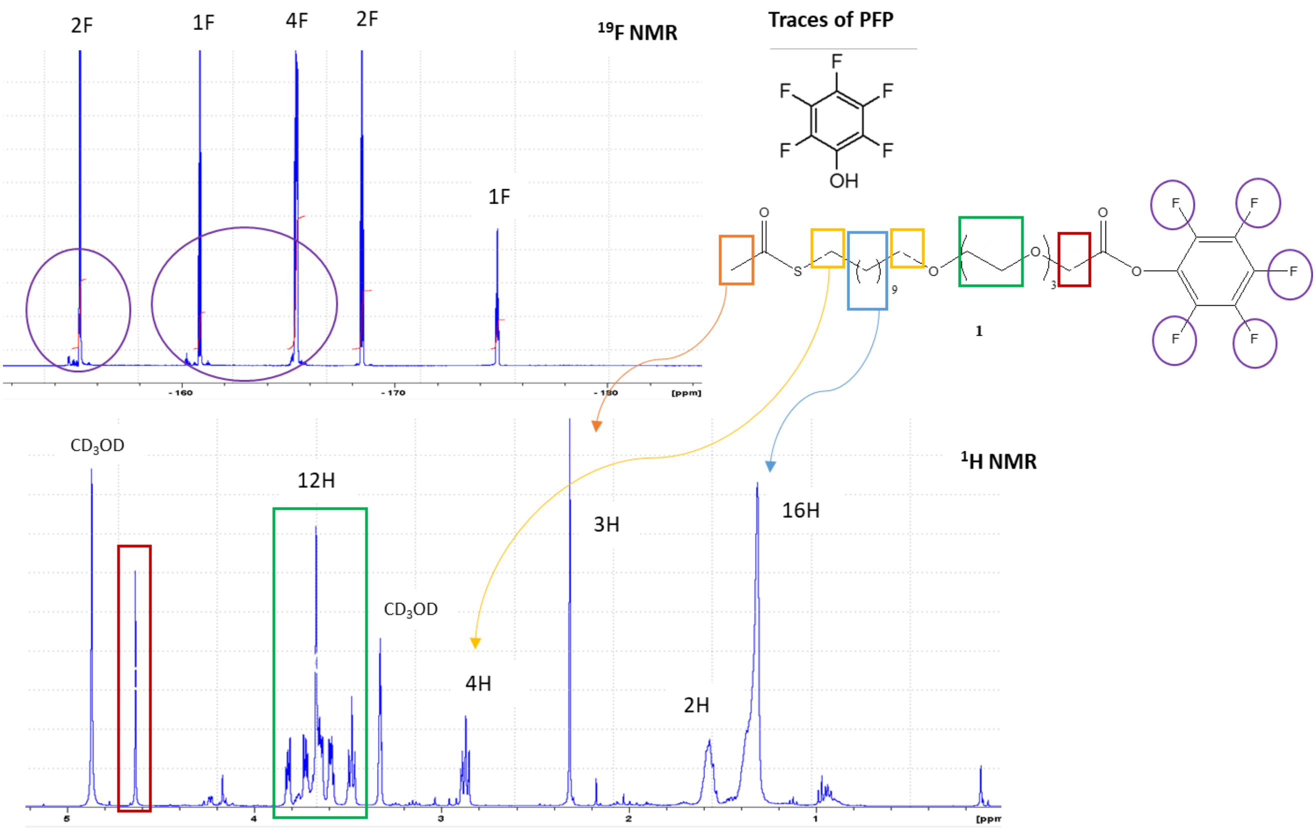

Figure S2 | ^{19}F and ^{1}H NMR spectra of PFP-EG$_6$C$_{11}$SH (400 MHz, methanol-d$_4$).

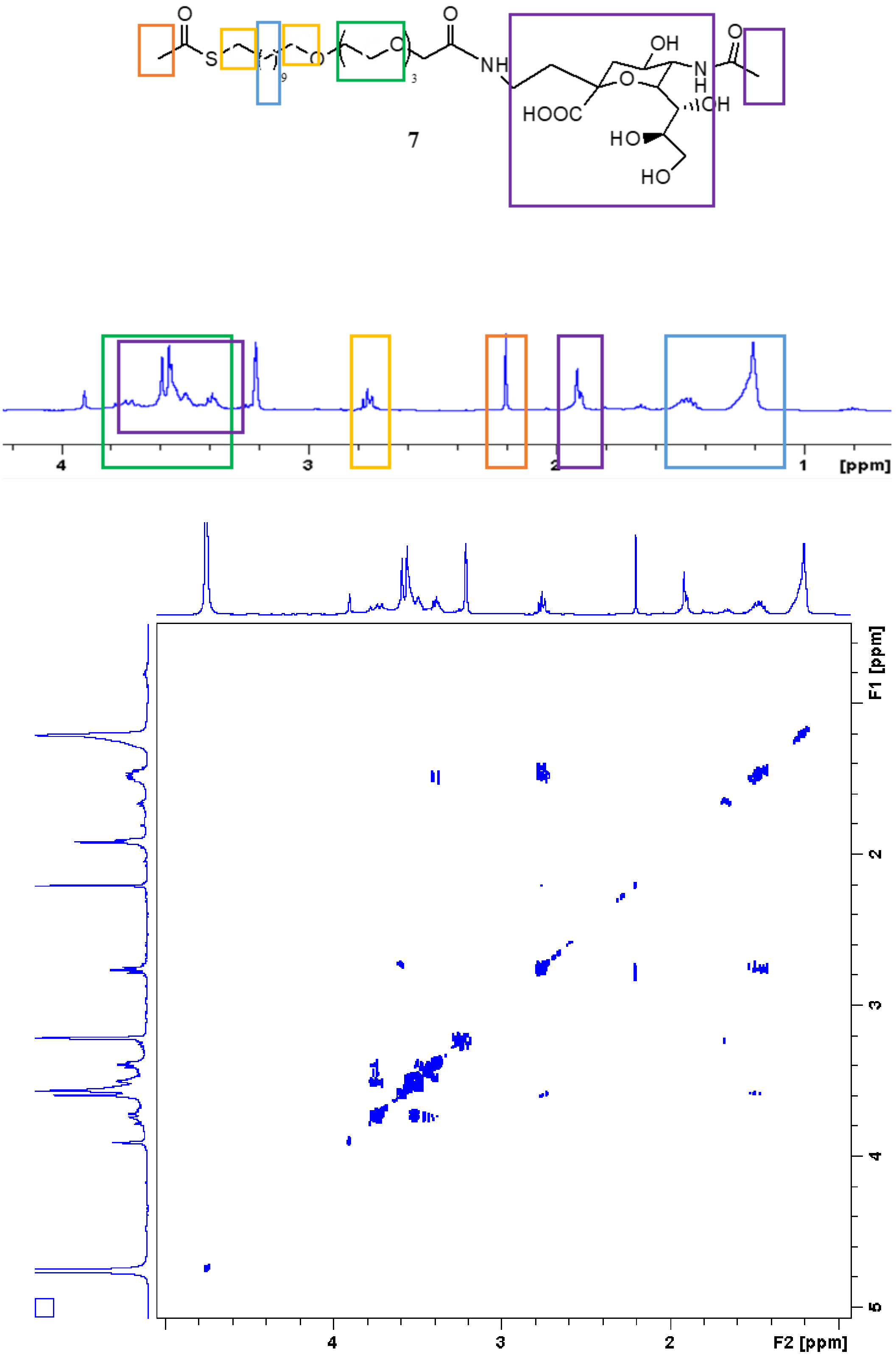

Figure S3 | ^{1}H NMR spectra and COSY of 3-aminopropyl-N-Acetylneuraminic acid-$EG_6C_{11}SH$ (400 MHz, methanol-d_4).

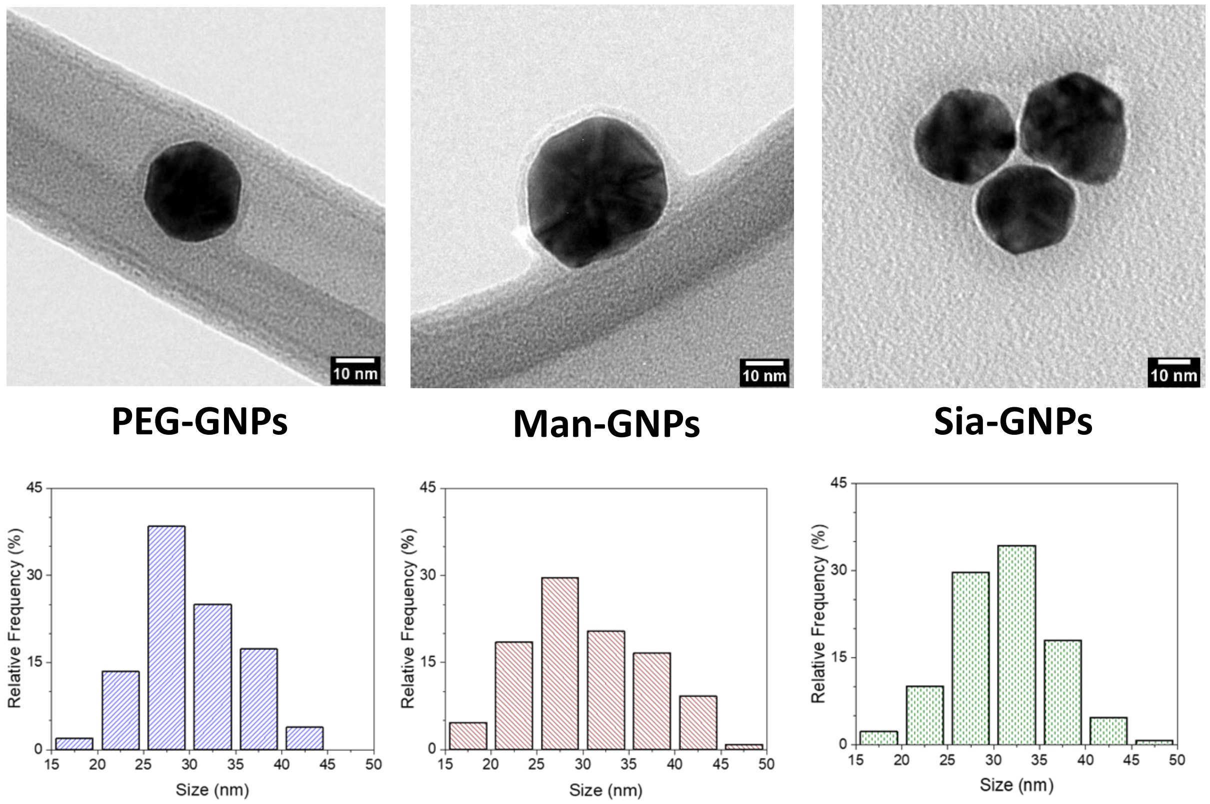

Figure S4 | TEM micrographs of glyco-GNPs with size distribution histograms. Scale bars for TEM images (small insets) = 10 nm. Size distribution histograms, after different steps of surface modifications, are plotted as relative frequency (%) that have a core diameter of dc. PEG-GNPs (dc = 30.2 ± 5.4 nm) (left), Man-GPs (dc = 30.3 ± 6.5 nm) (centre) and Sia-GNPs (dc = 30.9 ± 5.2 nm) (right).

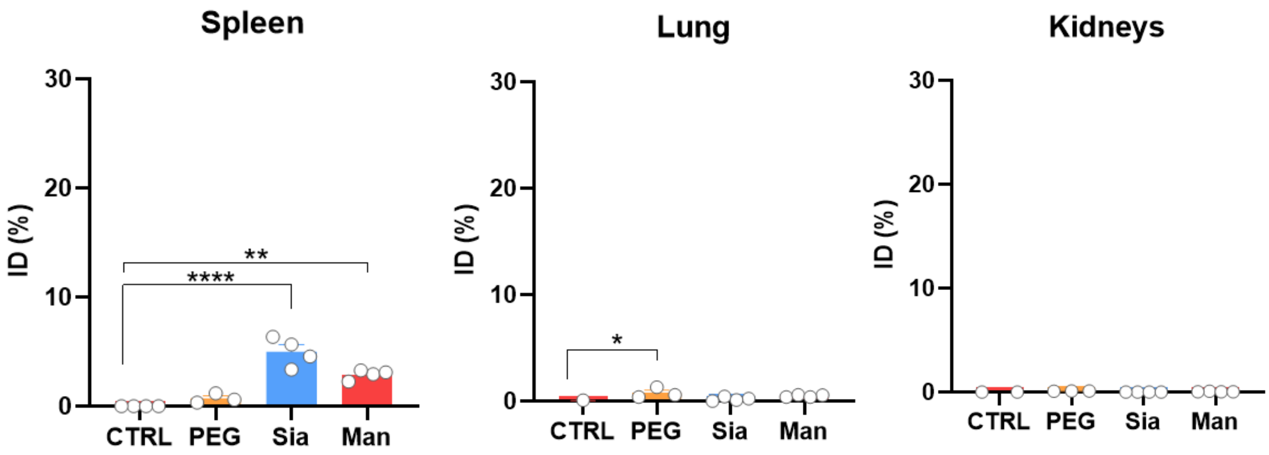

Figure S5 | ICP-MS of spleen, lung and kidneys after 4 of injection in healthy mice treated with glyco-GNPs. Data are presented as mean ± SEM. of n = 4 mice. P values were determined by one-way ANOVA with Bonferroni's correction *P < 0.05, **P < 0.01 and ****P < 0.0001.

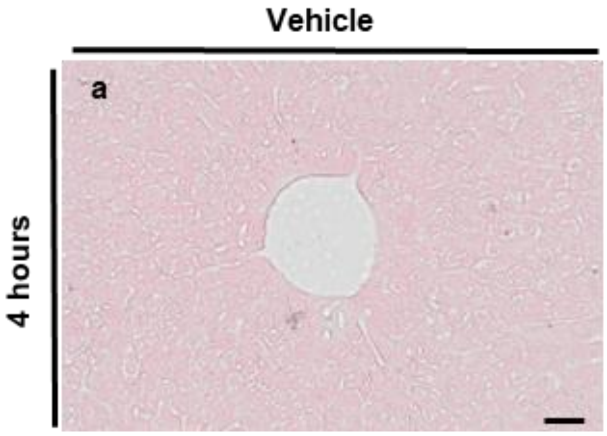

Figure S6 | Histological evaluation of gold distribution (black spots) in liver tissue by AMG. a Representative micrograph of liver from non-treated mice (vehicle). Scale bars for AMG images = 50 μm.

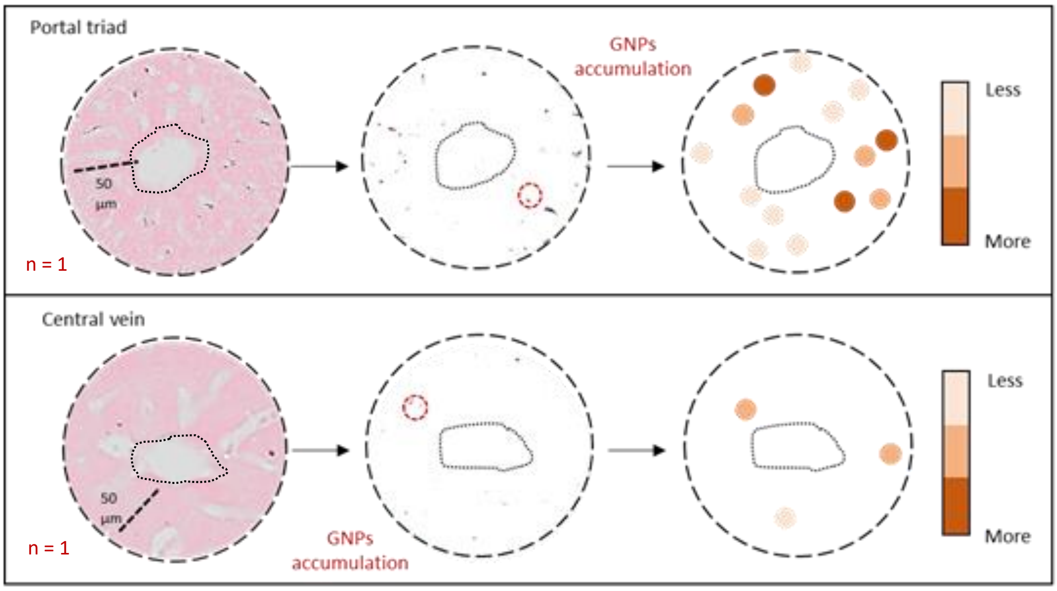

Figure S7 | Overview of the image processing used to measure the glyco-GNPs accumulation in the areas bordering the PT and CV. A radius of 50 µm from the vessel border was converted into a binary format to isolate reduced silver. The area of reduced silver corresponds to the amount of GNP accumulation and is represented by a colour spectrum, where pale brown indicates a small amount of GNP accumulation and dark brown indicates a large amount of glyco-GNPs accumulation in each individual location.

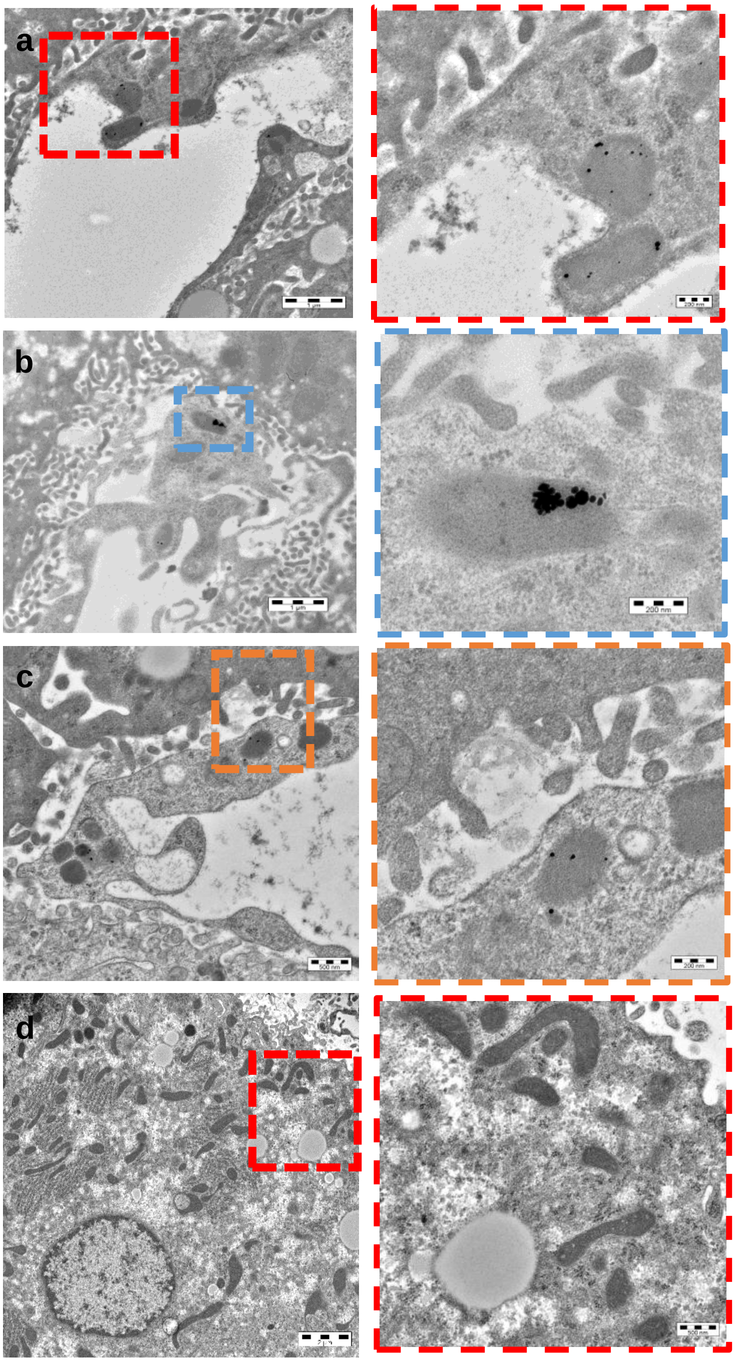

Figure S8 | Ultrastructural localization of glyco-GNPs inside hepatic cells by TEM. a-e) Representative TEM images of **a)** Man-GNPs in LSECs, **b)** Sia-GNPs in LSECs, **c)** PEG-GNPs in LSECs and **d)** hepatocytes with no GNPs in healthy mice treated after 4 hours. Images show GNPs internalized in lysosomes (small insets) of Kupffer cells and LSECs. Scale bars for TEM images (small insets) = 200 nm. Scale bars for TEM images (big insets) = 1 µm.

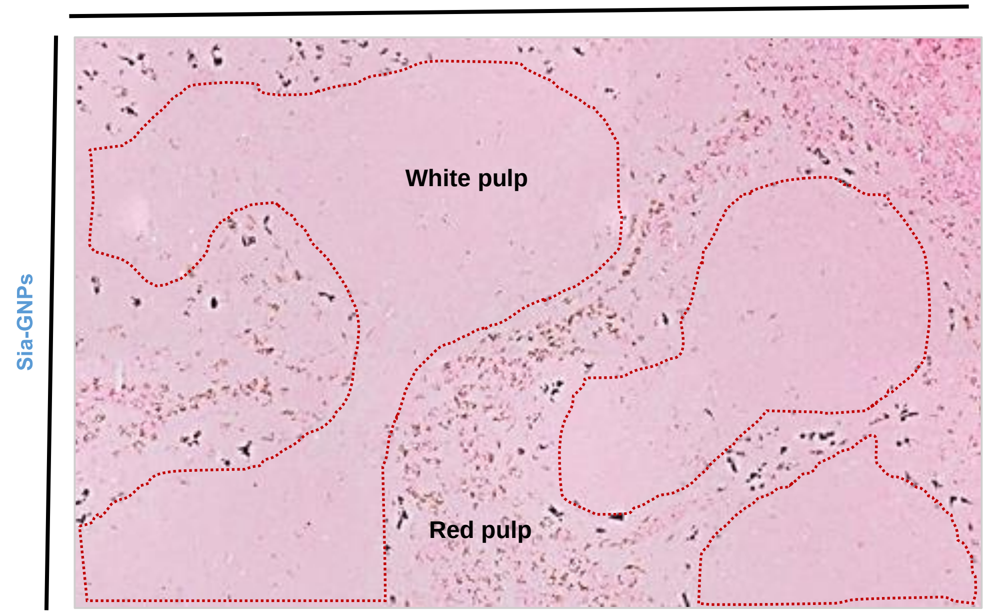

Figure S9 | Histological evaluation of gold distribution (black spots) in spleen tissue by AMG. a Representative micrograph of spleen treated with Sia-GNPs after 4h. Scale bars for AMG images = 50 μm.

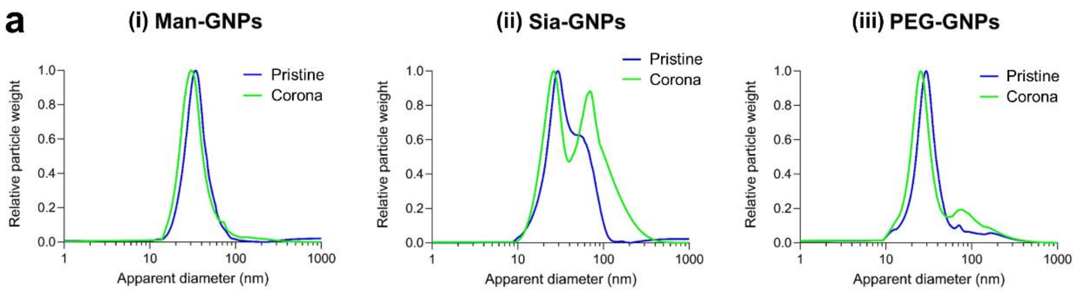

Figure S10 | Physico-chemical and biomolecular characterisations of the GNPs corona in mice serum. DCS analysis of the NP corona in situ after exposure to mice serum for (i) Man-GNPs, (ii) Sia-GNPs and (iii) PEG-GNPs.

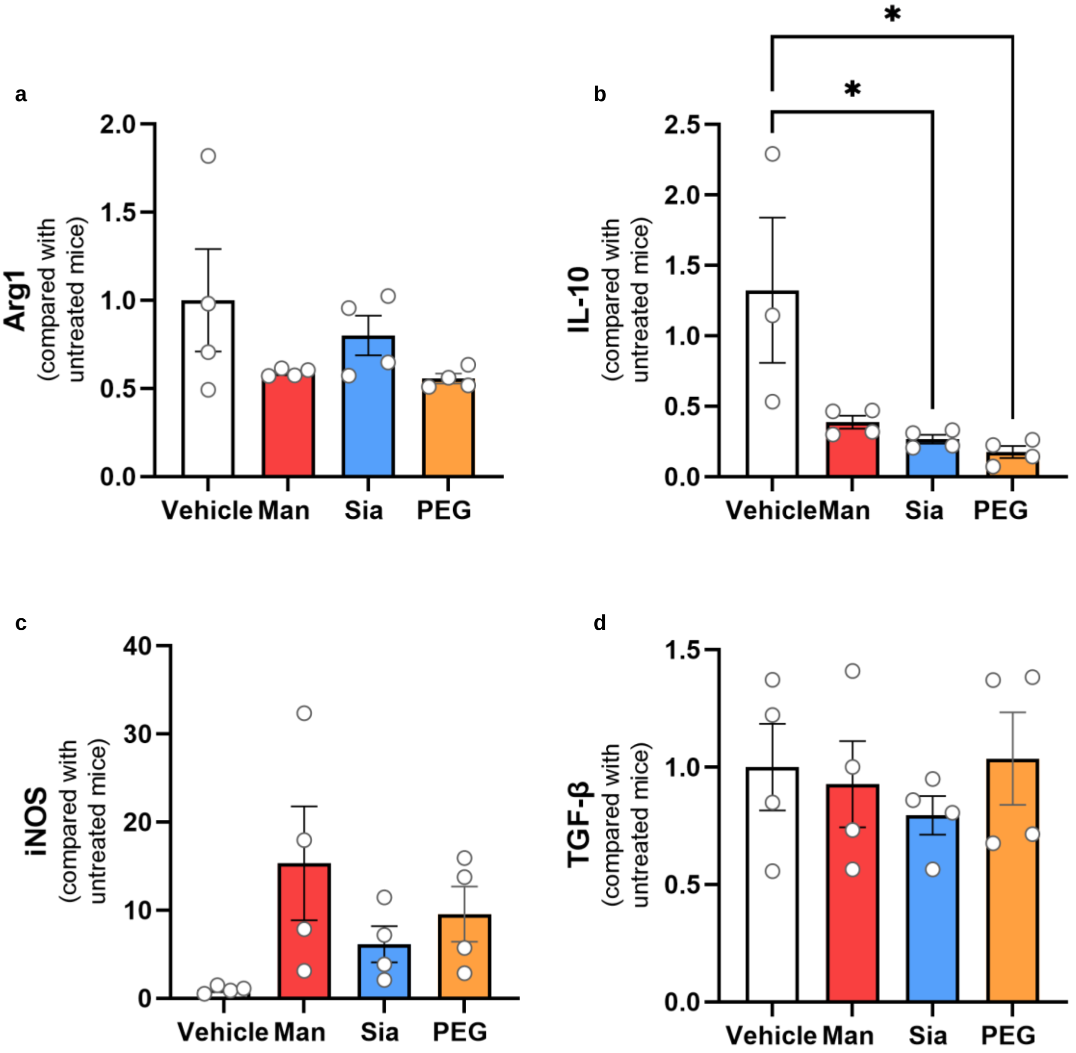

Figure S11 | rt-PCR of cytokines express in hepatic tissue of healthy mice treated with Man-GNPs, Sia-GNPs and PEG-GNPs sacrificed after 4 hours of treatment. Steady-state levels of mRNAs for all target genes were normalized to non-treated healthy mice (Vehicle). **a)** Gene expression of Arg-1 mRNAs, **b)** IL-10 mRNAs, **c)** iNOS mRNAs and **d)** TGF-β mRNAs. Data are presented as mean ± SEM. of n = 4 mice. P values were determined by one-way ANOVA with Bonferroni's correction *$P < 0.05$.

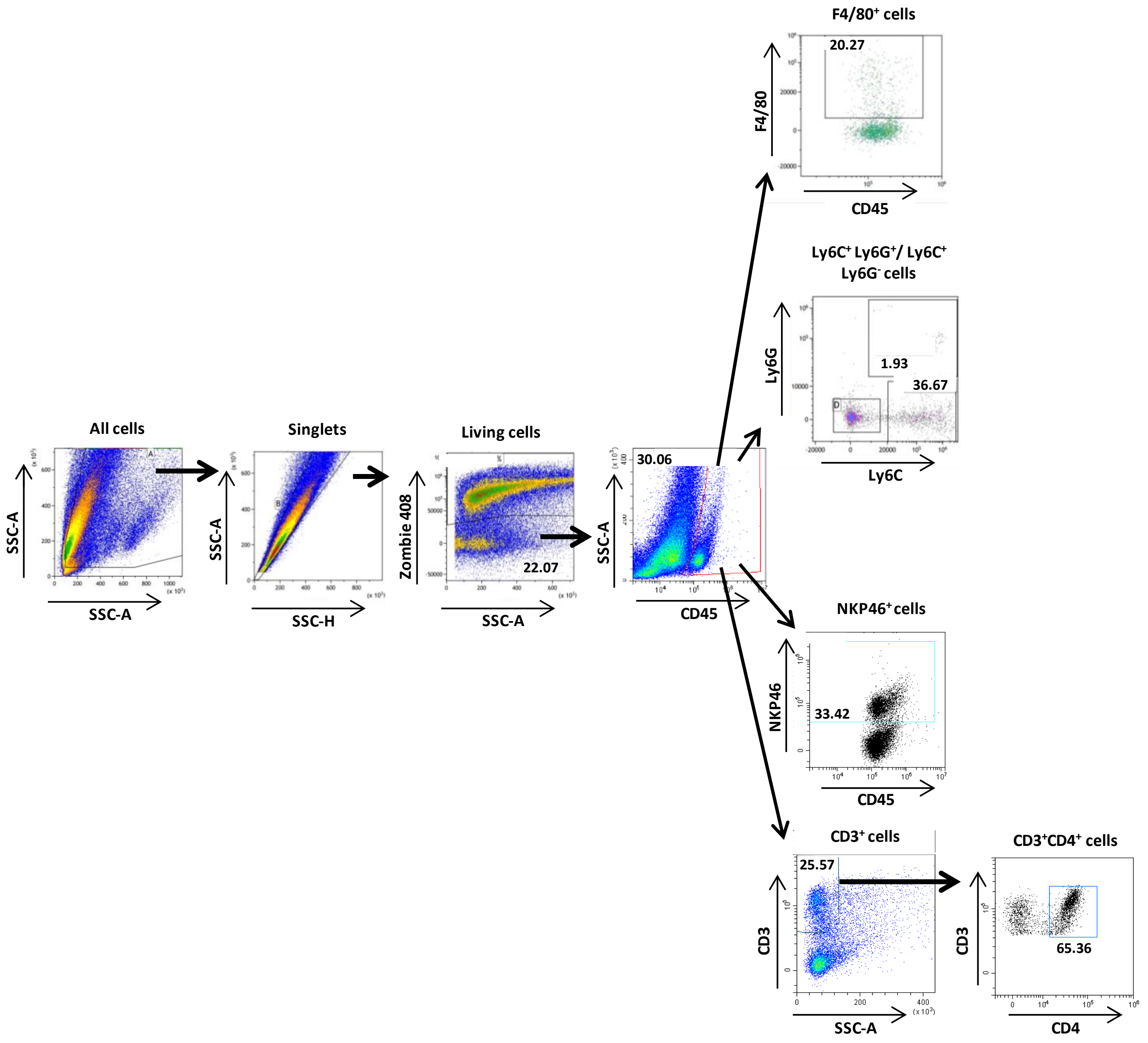

Figure S12 | Flow cytometry gating strategy. Gating strategy used to identify liver cell fractions in *in vivo* experiments after glyco-GNPs injection.

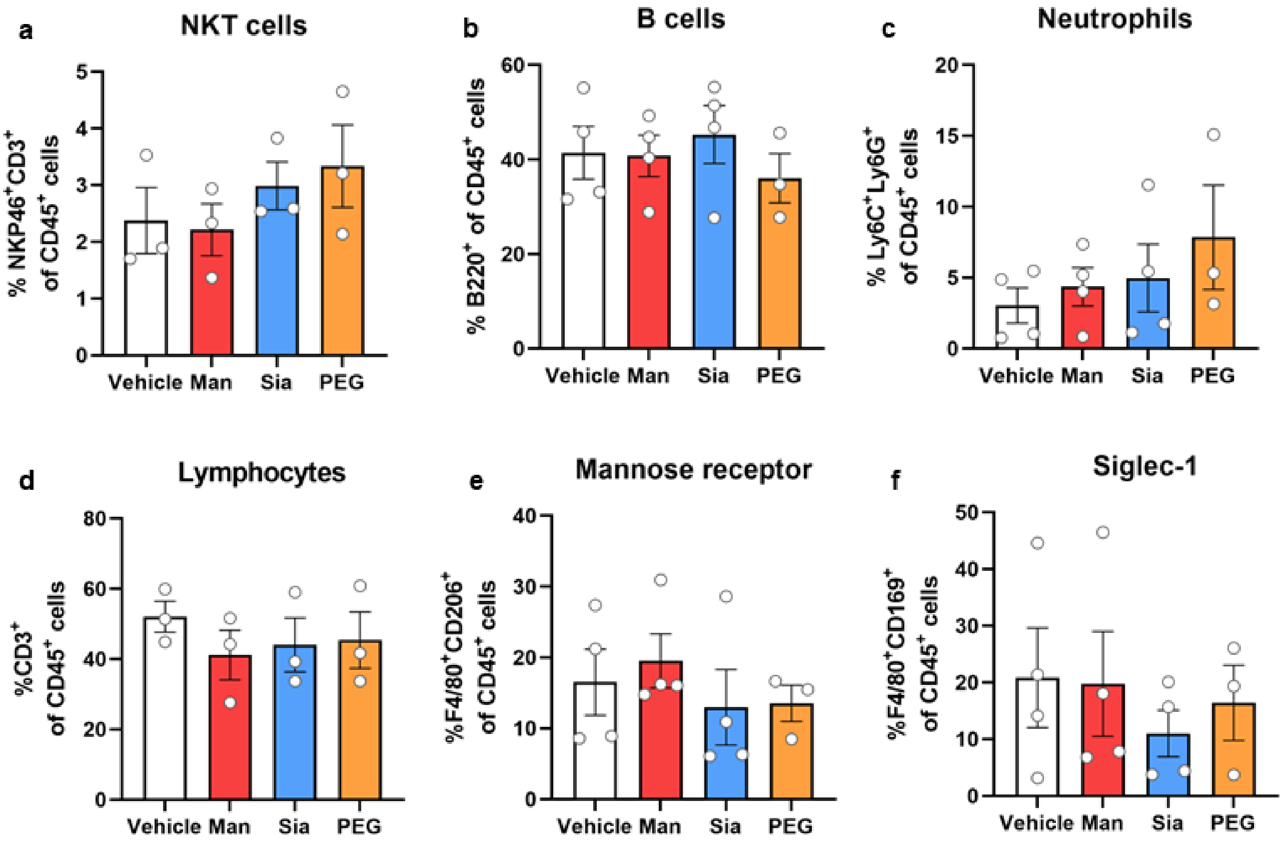

Figure S13 | Effect of a single dose administration of glyco-GNPs on the hepatic cell populations of healthy mice. a-f) Quantification of viable hepatic cell populations **a)** $CD45^+$ $NKP46^+$ $CD3^+$ T NK cells, **b)** $CD45^+$ $B220^+$ B cells, **c)** $CD45^+$ $Ly6C^+$ $Ly6G^+$ neutrophils, **d)** $CD45^+$ $CD3^+$ T cells, **e)** $CD45^+$ $F4/80^+$ $CD206^+$ macrophages, and **d)** $CD45^+$ $F4/80^+$ $CD169^+$ macrophages. Data are presented as mean ± SEM. of n = 3 mice. No statistical significance was found with $P > 0.05$.

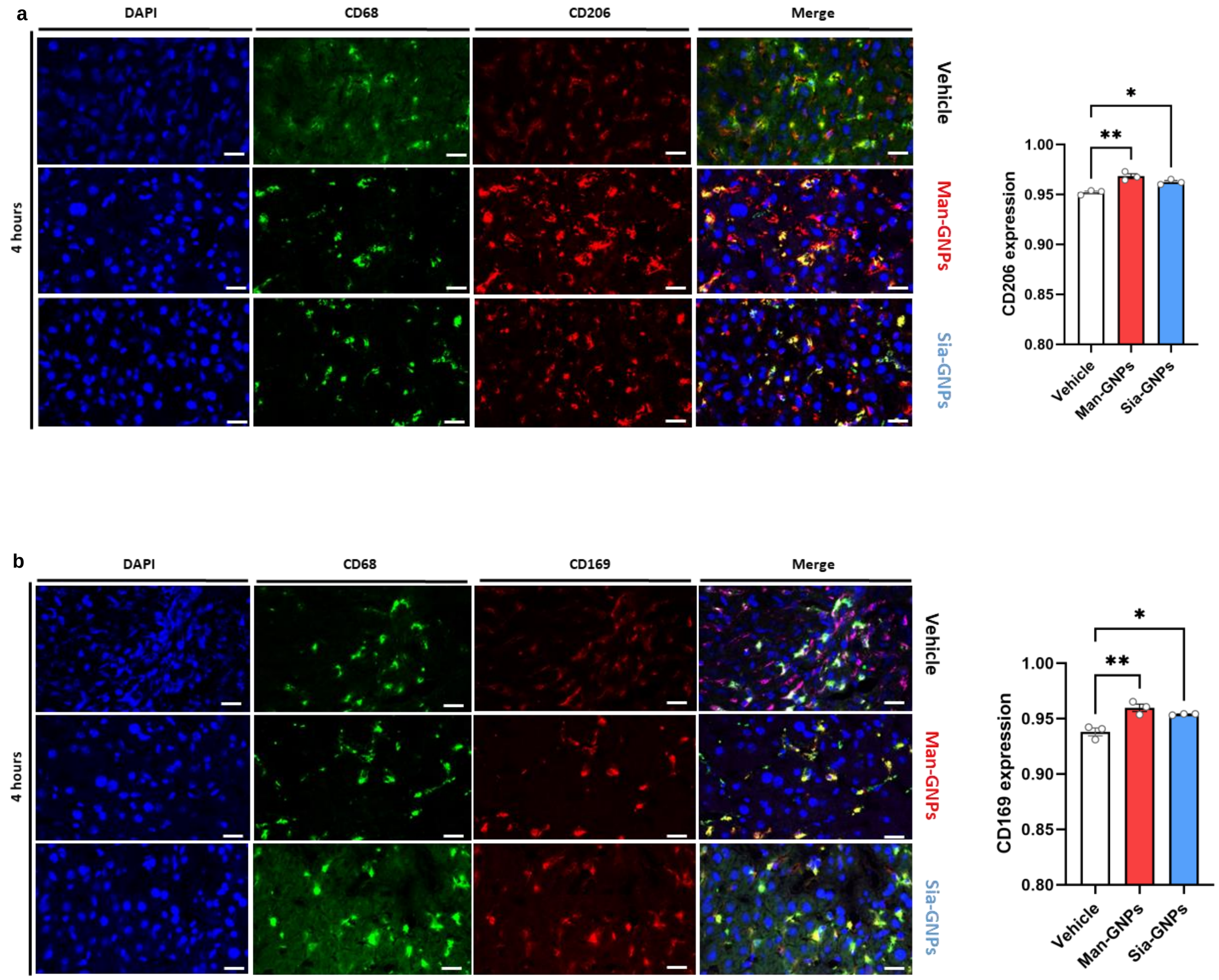

Figure S14 | Immunolabelling of hepatic tissues of healthy mice treated with Man-GNPs and Sia-GNPs. a) CD206 immunostaining and **b)** CD169 in livers of mice treated with Man-GNPs and Sia-GNPs after 4 hours. In blue, it was shown the nuclei (Hoescht), in red $CD206^+$ cells or $CD169^+$ cells, and in yellow co-localization. Scale bars = 25 μm. Quantification of $CD206^+$ and $CD169^+$ cells in hepatic tissue. Data are presented as mean ± SEM. of n = 3 mice. P values were determined by one-way ANOVA with Bonferroni's correction *$P < 0.05$, and **$P < 0.01$

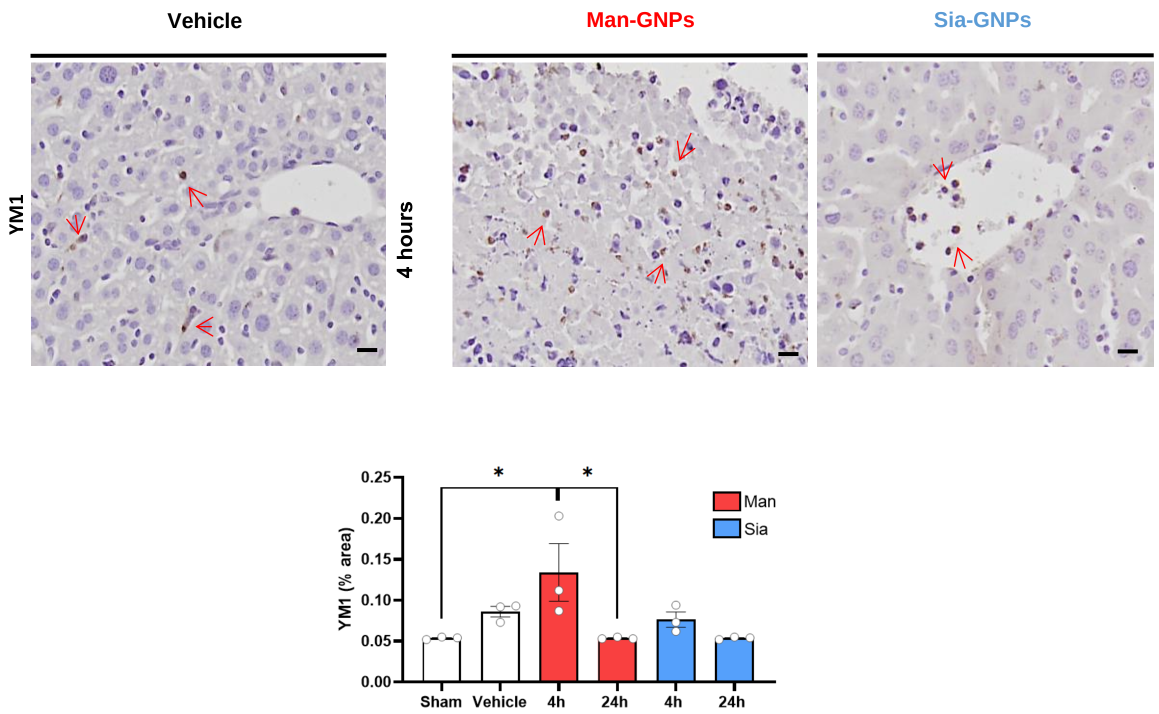

Figure S15 | Histological evaluation of metastatic hepatic tissues of tumor bearing mice of CRC treated with Man-GNPs and Sia-GNPs. a) Representative micrographs of liver from mice treated with Man-GNPs and Sia-GNPs sacrificed after 4 hours by YM1 staining. Hematoxylin stains the cell nuclei in blue and in brown the macrophages. Red arrows show $YM1^+$ cells in liver. Scale bars for YM1 images = 20 µm. Quantification of $YM1^+$ cells in hepatic tissue. Data are presented as mean ± SEM. of n = 3 mice. P values were determined by one-way ANOVA with Bonferroni's correction *$P < 0.05$.

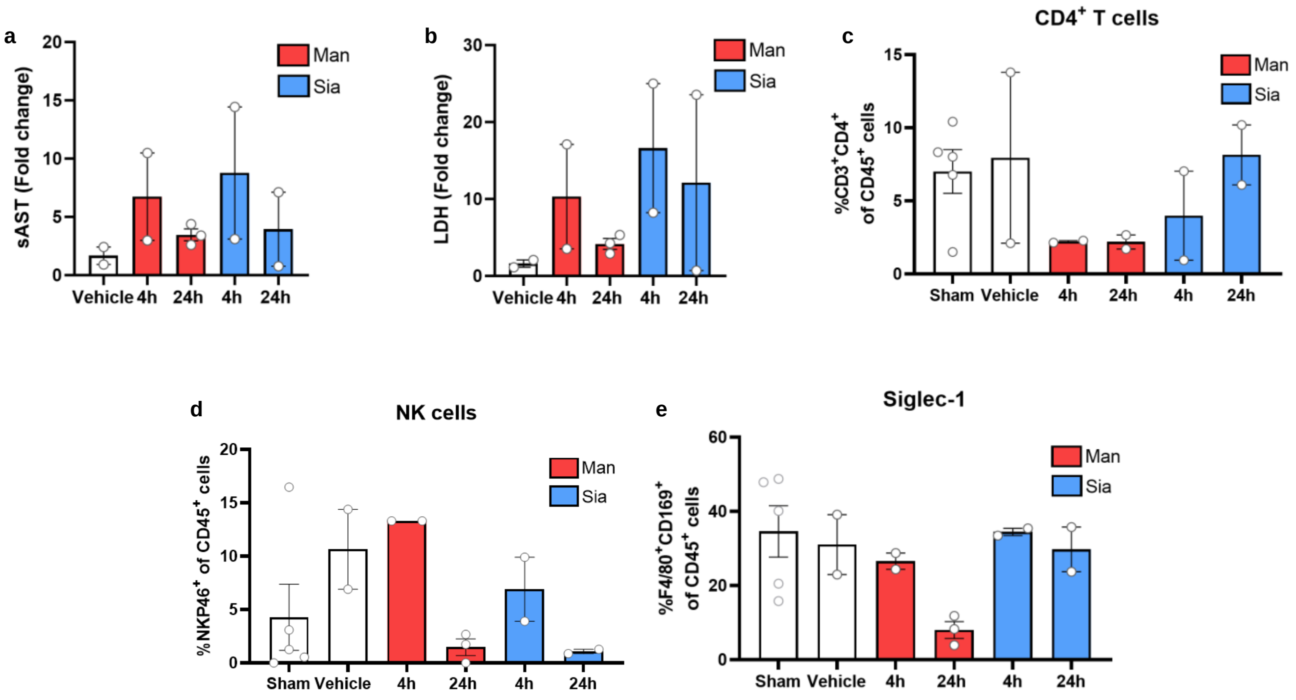

Figure S16 | Effect of a single dose administration of glyco-GNPs on the hepatic cell populations of tumor bearing mice CRC with hepatic metastasis. a-b) Hepatic transaminases levels in serum. Fold change of sAST and sLDH measured after 4 and 24 hours in groups of mice injected with Man-GNPs and Sia-GNPs. Data are presented as mean ± SEM. of n = 2 mice. No significant difference was determined P >0.05. **c-e)** Quantification of viable hepatic cell populations **c)** $CD45^+$ $CD3^+$ $CD4^+$ T cells, **d)** $CD45^+$ $NKP46^+$ NK cells, and **e)** $CD45^+$ $F4/80^+$ $CD169^+$ macrophages. Data are presented as mean ± SEM. of n = 3 mice. No statistical significance was found with P > 0.05.

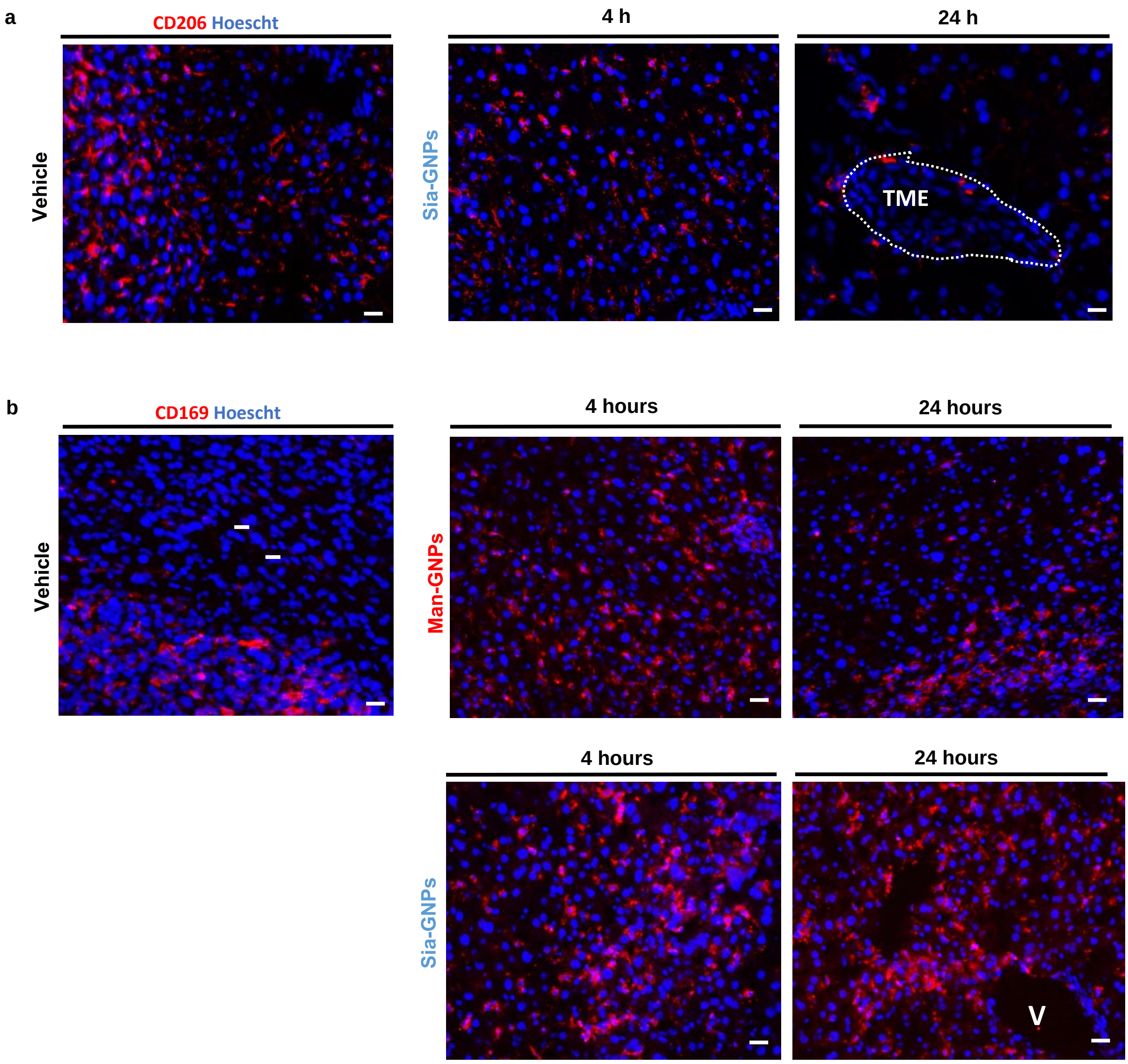

Figure S17 | Immunolabelling of metastatic hepatic tissues of tumor bearing mice of CRC treated with Man-GNPs and Sia-GNPs. a) CD206 immunostaining in livers of mice treated with Man-GNPs and Sia-GNPs after 24 hours. In blue, it was shown the nuclei (Hoescht) and in red CD206$^+$ cells. **b)** CD169 immunostaining in livers of mice treated with Man-GNPs and Sia-GNPs after 4 and 24 hours and vehicle mice. In blue, it was shown the nuclei (Hoescht) and in red CD169$^+$ cells. (V) Indicates vessel. Scale bars = 25 µm.

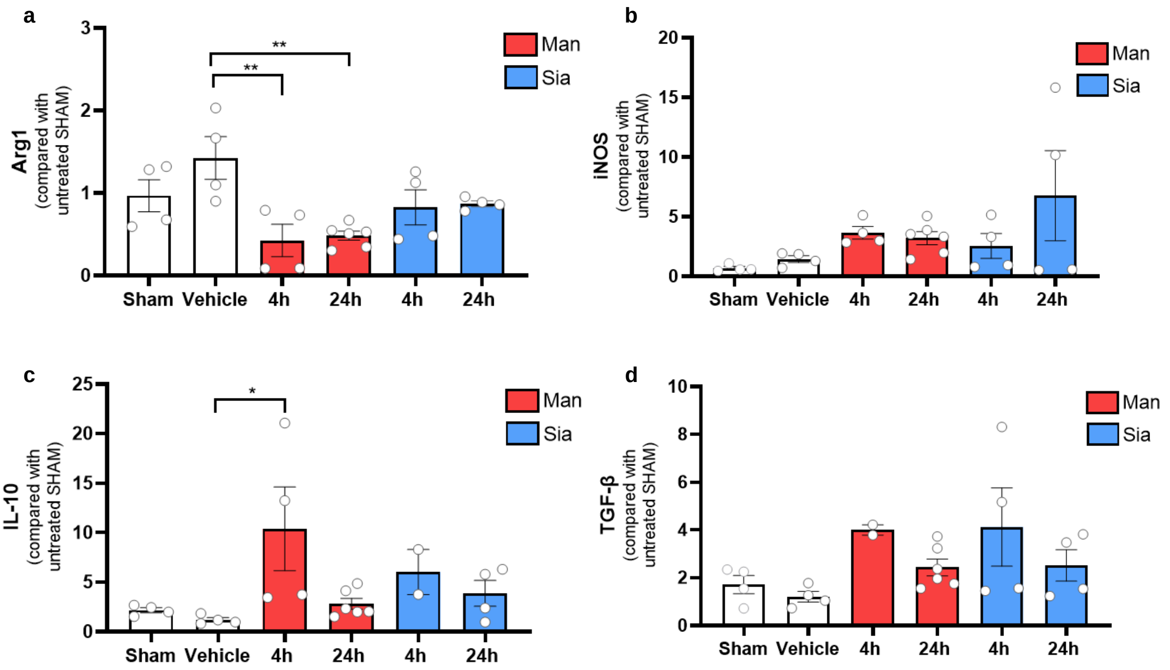

Figure S18 | rt-PCR of cytokines express in hepatic tissue of tumor bearing mice with CRC treated with Man-GNPs and Sia-GNPs sacrificed after 4 and 24 hours of treatment. Steady-state levels of mRNAs for all target genes were normalized to non-treated sham mice. **a)** Gene expression of Arg1 mRNAs, **b)** iNOS mRNAs, **c)** IL-10 mRNAs and **d)** TGF-β mRNAs. Data are presented as mean ± SEM. of n = 2 mice. P values were determined by one-way ANOVA with Bonferroni's correction *P < 0.05 and **P < 0.01.

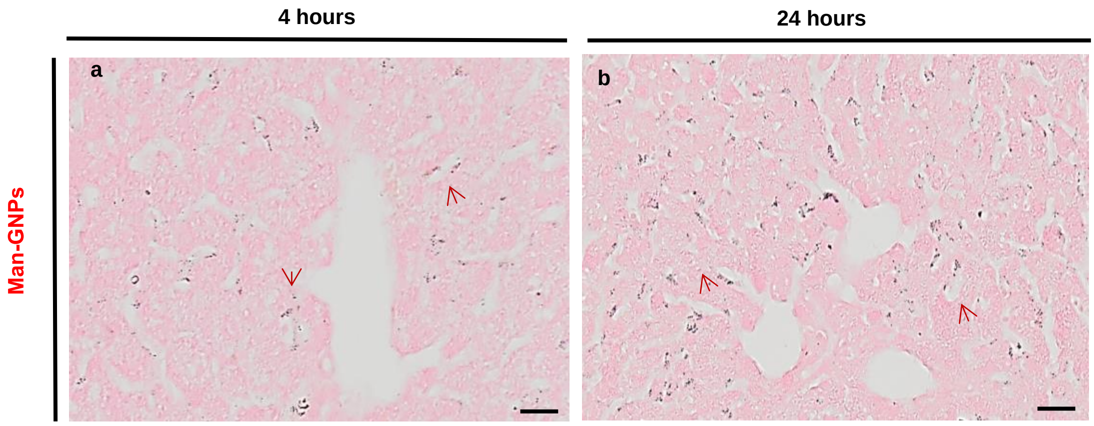

Figure S19 | Histological evaluation of gold distribution (black spots) in liver tissue by AMG in PBC model, female ARE Del$^{-/-}$ mice. a-b) Representative micrographs of liver stained with AMG from mice treated with Man-GNPs euthanized after **a)** 4 and **b)** 24 hours by AMG (black spots). Red arrows show silver-stained glyco-GNPs inside hepatic cells. Scale bars = 100 μm.

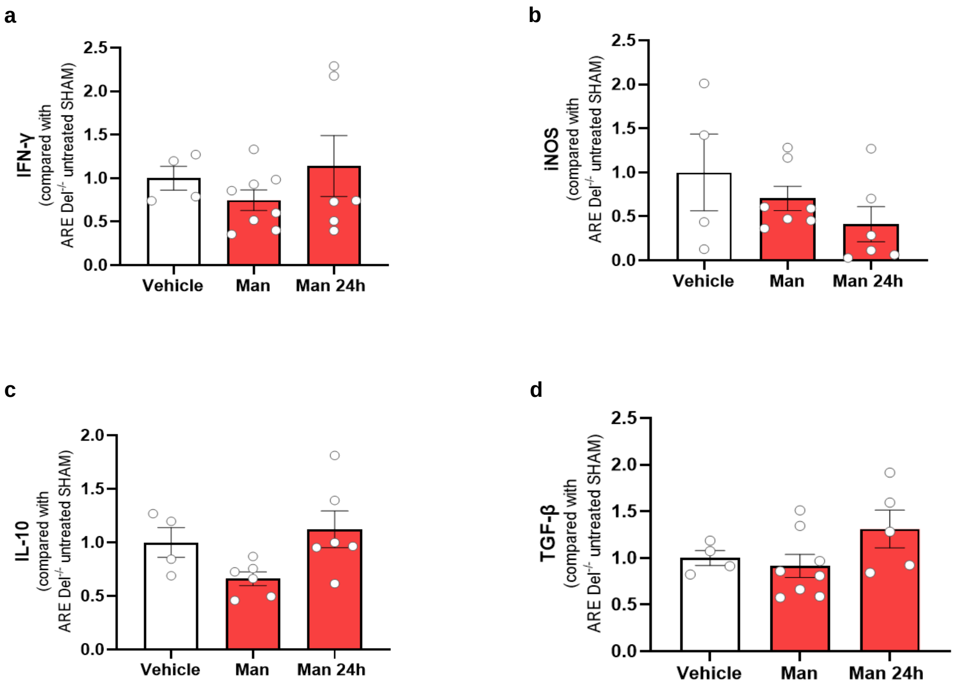

Figure S20 | rt-PCR of cytokines express in hepatic tissue of ARE Del$^{-/-}$ mice treated with Man-GNPs sacrificed after 4 and 24 hours of treatment. Steady-state levels of mRNAs for all target genes were normalized to non-treated sham mice. **a)** Gene expression of IFN-γ mRNAs, **b)** iNOS mRNAs, **c)** IL-10 mRNAs and **d)** TGF-β mRNAs. Data are presented as mean ± SEM. of n = 3 mice. No statistical significance was found with $P > 0.05$.

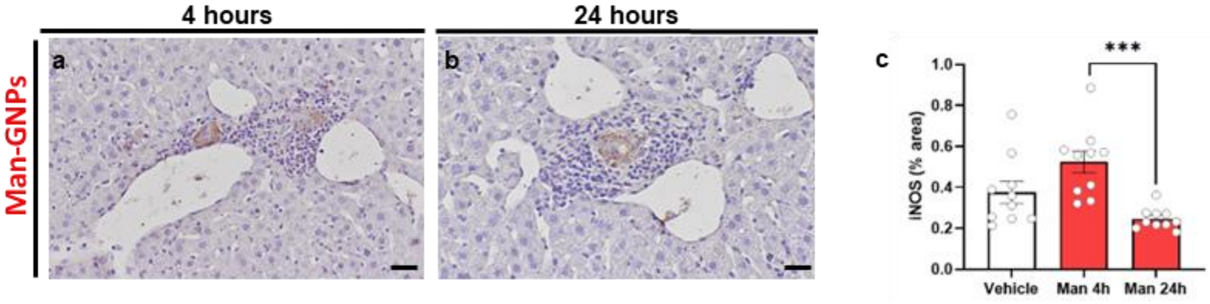

Figure S21 | Histological evaluation of PBC model, ARE Del$^{-/-}$ mice treated with Man-GNPs. a-b) Tissue sections of liver stained by iNOS, from treated ARE Del$^{-/-}$ mice 18 weeks old and sacrificed after **a)** 4 and **b)** 24 hours. Hematoxylin stains the cell nuclei in blue and in brown the macrophages with a pro-inflammatory phenotype. Scale bars = 50 μm. **c)** Quantification of iNOS^{+} cells in hepatic tissue. Data are presented as mean ± SEM. of n = 3 mice. P values were determined by one-way ANOVA with Bonferroni's correction ***P < 0.001.

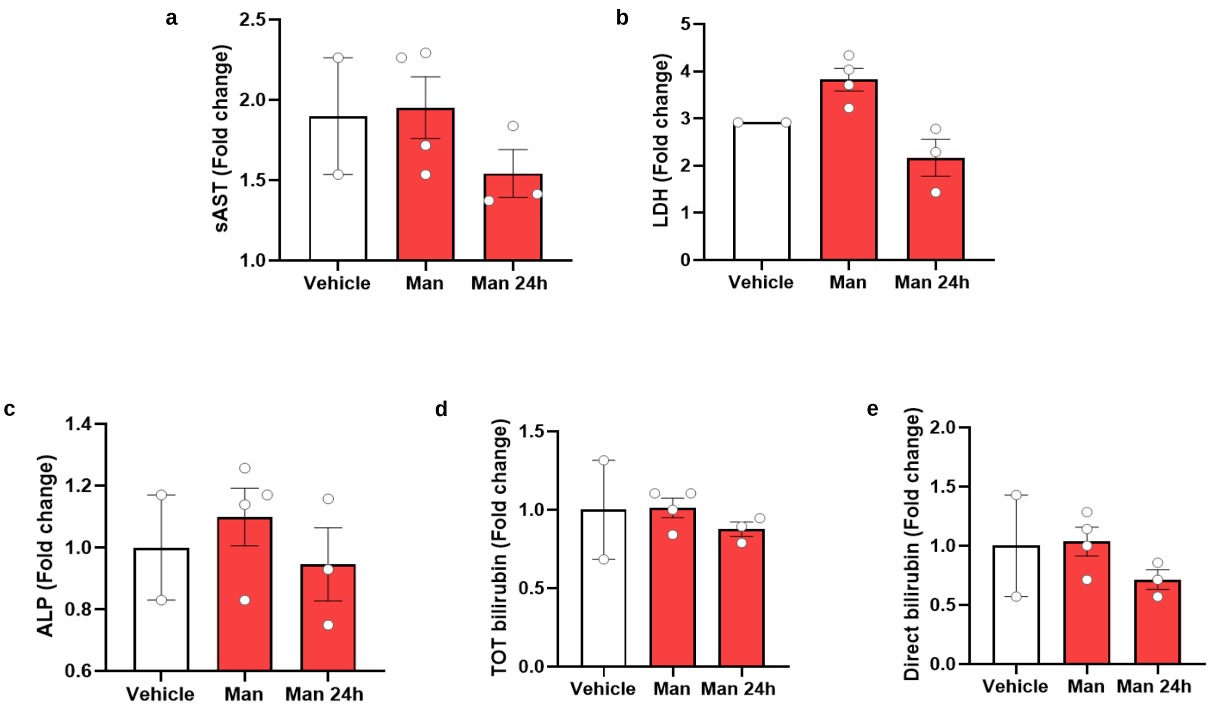

Figure S22 | Effect of a single dose administration of Man-GNPs on the hepatic transaminases levels of ARE Del$^{-/-}$ mice. a-e) Hepatic transaminases levels in serum. Fold change of **a)** sAST, **b)** sLDH, **c)** sALP, **d)** total bilirubin, and **e)** direct bilirubin, measured after 4 and 24 hours in groups of mice injected with Man-GNPs. Data are presented as mean ± SEM. of n = 3 mice. No significant difference was determined P >0.05.

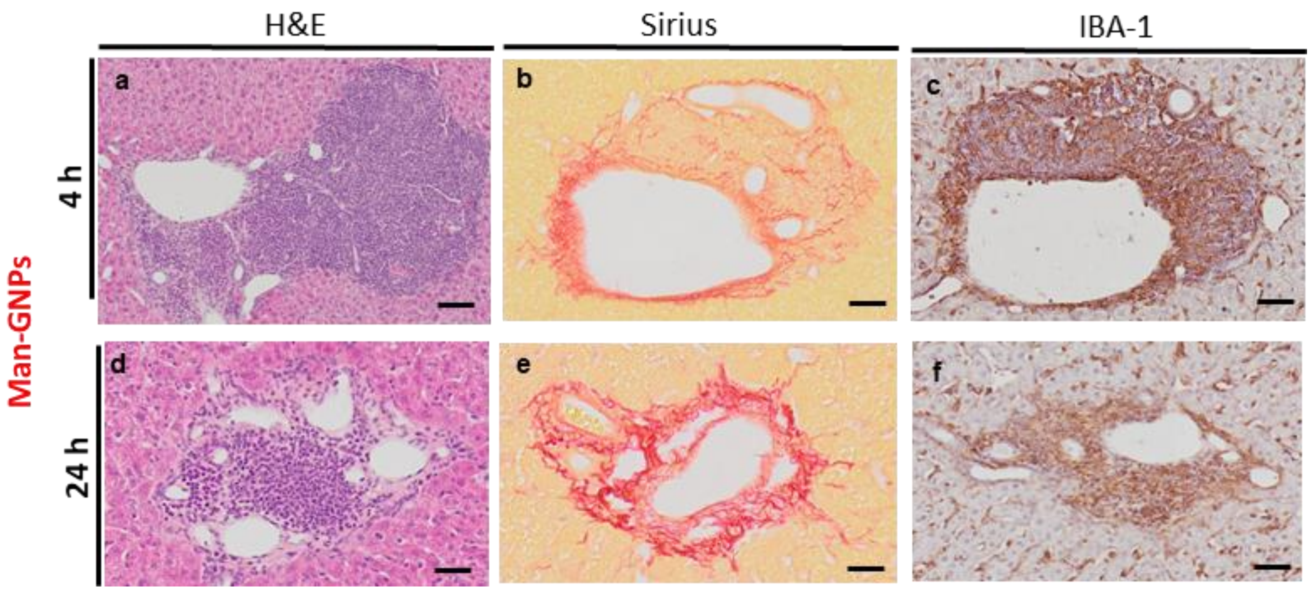

Figure S23 | Histological evaluation of PBC model, male ARE Del$^{-/-}$ mice treated with Man-GNPs. a-f) Tissue sections of liver from ARE Del$^{-/-}$ mice 18 weeks old sacrificed after **a-c)** 4 and **d-f)** 24 hours with three different histological stains: **a-d** H&E, hematoxylin stains the cell nuclei in blue and eosin stains the extracellular matrix and cytoplasm in pink. **b-e** Sirius red, most of the tissue is stained in yellow and in red the collagen fibers that surround hepatic veins and sinusoids and **c-f** IBA-1, hematoxylin stains the cell nuclei in blue and in brown the macrophages. Scale bars = 50 μm.

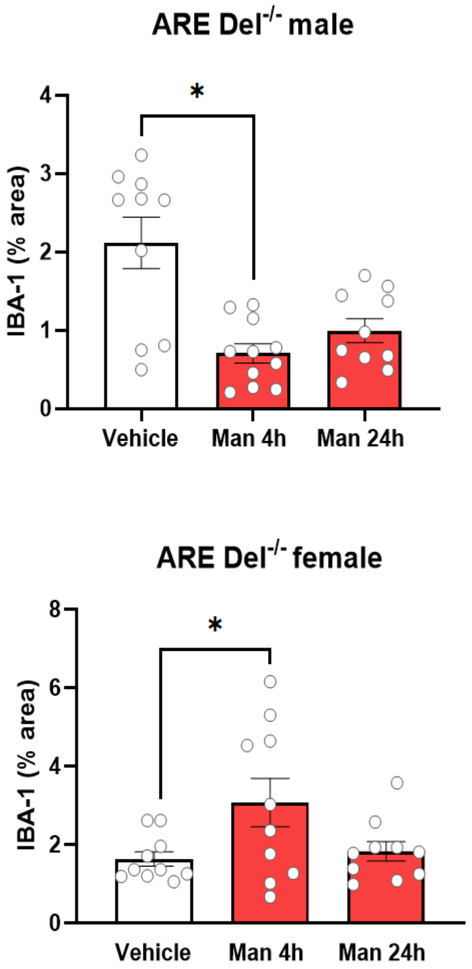

Figure S24 | Quantification of IBA1$^+$ cells in histological tissues of male and female ARE Del$^{-/-}$ mice treated with Man-GNPs. Quantification of IBA1$^+$ cells in hepatic tissue. Data are presented as mean ± SEM. of n = 3 mice. P values were determined by one-way ANOVA with Bonferroni's correction *P < 0.05.

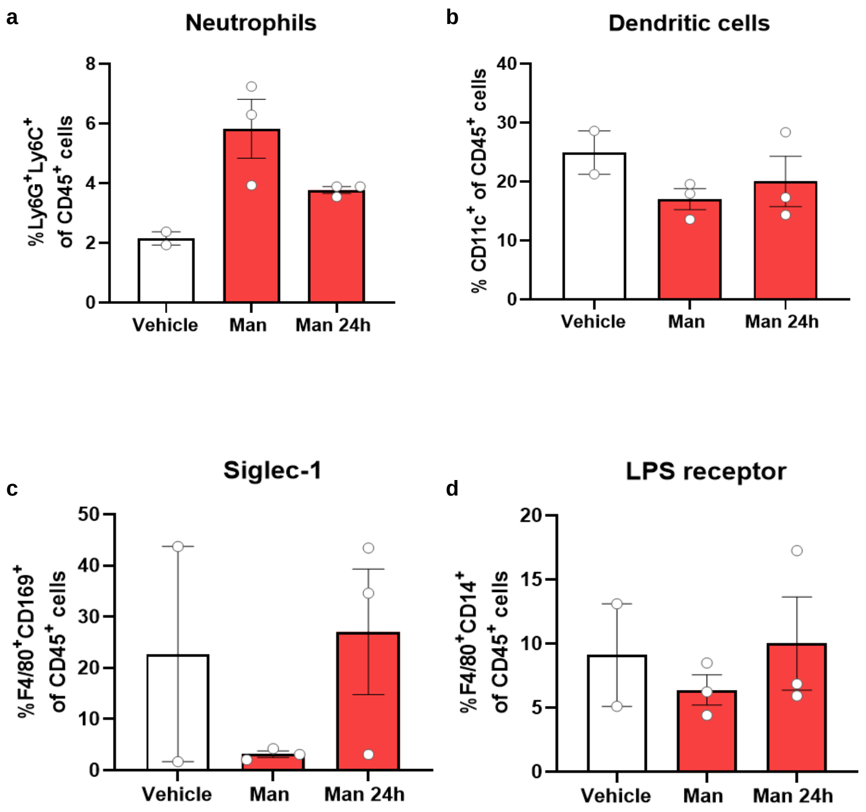

Figure S25 | Effect of a single dose administration of Man-GNPs on the hepatic cell populations of ARE Del$^{-/-}$ mice. a-d) Quantification of viable hepatic cell populations **a)** $CD45^+Ly6C^{high}Ly6G^+$ neutrophils, **b)** $CD45^+$ $CD11b^+$ dendritic cells, **c)** $CD45^+$ $F4/80^+$ $CD169^+$ macrophages, and **d)** $CD45^+$ $F4/80^+$ $CD14^+$ macrophages. Data are presented as mean ± SEM. of n = 3 mice. No statistical significance was found with $P > 0.05$.

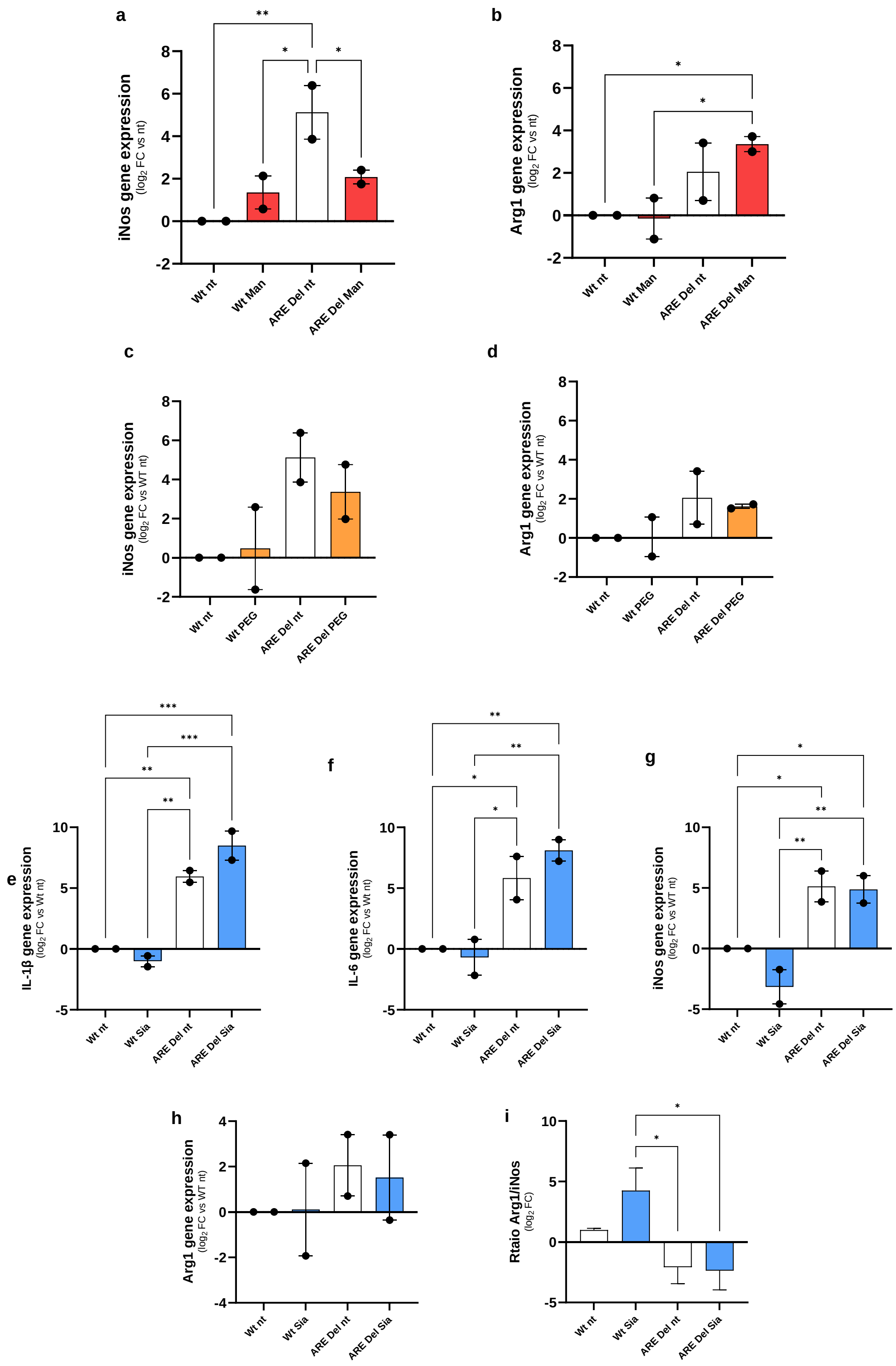

Figure S26 | rt-PCR of cytokines express in KCs from ARE Del$^{-/-}$ mice treated with Man-GNPs, PEG-GNPs and Sia-GNPs after 24 hours of treatment. a-b) Gene expression of iNOS and Arg1 mRNAs treated with Man-GNPs, **c-d)** gene expression of iNOS and Arg1 mRNAs treated with PEG-GNPs and, **e-i)** gene expression of IL-1β, IL-6, iNOS and Arg1 mRNAs treated with Sia-GNPs. Data are presented as mean ± SEM. of n = 2 mice. P values were determined by one-way ANOVA with Bonferroni's correction *P < 0.05, P < 0.01 and, ***P < 0.001.

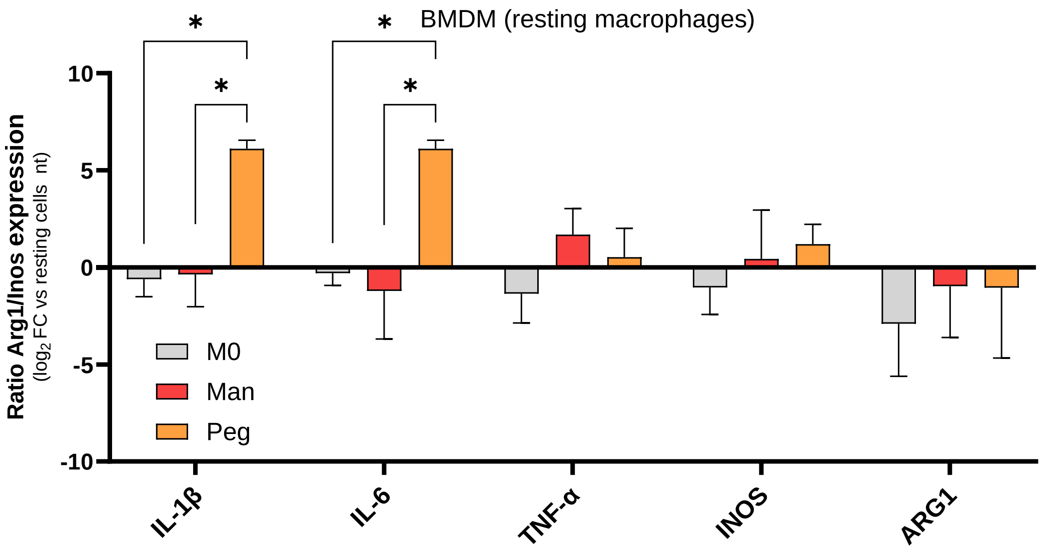

Figure S27 | rt-PCR of cytokines express in BMDMs treated with Man-GNPs and PEG-GNPs after 24 hours of treatment. Gene expression of ratio Arg1/iNOS. Data are presented as mean ± SEM. of n = 3. P values were determined by one-way ANOVA with Bonferroni's correction *$P < 0.05$.

Gene name	Application	Sequence
β-actin	rt-PCR	fw GCCCTGAGGCTCTTTTCCAG
β-actin	rt-PCR	rv TGCCACAGGATTCCATACCC
TNF-α	rt-PCR	fw AGACCCTCACACTCAGATCATCTTC
TNF-α	rt-PCR	rv TTGCTACGACGTGGGCTACA
IL-1β	rt-PCR	fw AGTTGACGGACCCCAAAAGA
IL-1β	rt-PCR	rv GGACAGCCCAGGTCAAAGG
IL-6	rt-PCR	fw TCTCTGGGAAATCGTGGAAA
IL-6	rt-PCR	rv TCTGCAAGTGCATCATCGTT
Arg1	rt-PCR	fw CATGGGCAACCTGTGTCCTT
Arg1	rt-PCR	rv TCCTGGTACATCTGGGAACTTTC
IL-10	rt-PCR	fw TGATGGGAGGGGTTCTTCCT
IL-10	rt-PCR	rv AGGACACCATAGCAAAGGGC
TGF-β	rt-PCR	fw CCAAGGACACGGAATACAGGG
TGF-β	rt-PCR	rv TCACAAGAGCAGTGAGCGCT
iNOS	rt-PCR	fw CAAGCACCTTGGAAGAGGA
iNOS	rt-PCR	rv AAGGCCAAACACAGCATACC

Supplementary table 1. List of primers used in this study.

References

[1] Á. G. Barrientos, J. M. de la Fuente, T. C. Rojas, A. Fernández, S. Penadés, *Chem. Eur. J.* **2003**, *9*, 1909.
